# Folate-Functionalized Albumin-Containing Systems: Non-Covalent vs. Covalent Binding of Folic Acid

**DOI:** 10.3390/pharmaceutics18010054

**Published:** 2025-12-31

**Authors:** Maria G. Gorobets, Anna V. Toroptseva, Madina I. Abdullina, Derenik S. Khachatryan, Anna V. Bychkova

**Affiliations:** Emanuel Institute of Biochemical Physics of Russian Academy of Sciences, 4, Kosygina Street., Moscow 119334, Russia; stropdiva@yandex.ru (A.V.T.); triyozhika@gmail.com (M.I.A.); derenik-s@yandex.ru (D.S.K.)

**Keywords:** folic acid, folate-albumin conjugation, NHS-ester, albumin, nano- and submicron particles (NSPs), conjugation, covalent binding, non-covalent binding, carbodiimide reaction

## Abstract

Nano- and submicron particles (NSPs) with folate for targeting are actively used for the treatment and diagnosis of cancer and inflammatory diseases. Albumin-containing systems have enhanced biocompatibility, circulation time, and colloidal stability, which are important for medical applications. The outstanding binding properties of albumin allow the transport of numerous therapeutic and/or imaging agents. This review summarizes multiple aspects of binding a folate residue (or folic acid) to NSPs and the functioning of folate-albumin-NSPs. Special attention in the review is given to the types of bonds between folic acid and albumin, i.e., covalent and non-covalent, and to the confirmation and quantification of binding by different physicochemical methods. The process of binding, the qualitative and quantitative characteristics of binding and forming product, and its functioning are interconnected with the binding conditions; thus, an analysis of reaction conditions is provided. For the proper functioning of folate-albumin-NSPs, the state of albumin within them is important; thus, considerable focus in the review is placed on the features of structure modification of serum albumin in folate-albumin binding, i.e., the amino acid residues involved in this process and the conformational state of the protein. The stability and the functioning of the protein within folate-albumin-NSPs are discussed. Also, the effectiveness of targeting by folate is viewed as dependent on many characteristics of folate-albumin-NSPs, particularly on the peculiarities of binding between the folic acid residue and albumin. Furthermore, the authors discussed and suggested solutions concerning the shortcomings highlighted in the studies devoted to obtaining folate-modified albumin-containing NSPs.

## 1. Introduction

Folic acid (FA) is a well-known molecule that has been used for the treatment and prevention of cardiovascular diseases, hematopoietic disorders, and neurological diseases, etc. [1,2,3,4]. Folate, as a vitamin needed for the biosynthesis of nucleotide bases and cell proliferation, enters through folic acid (or folate) receptors (FR) to all cells of living organisms, but it is known that FRs are overexpressed on the surface of some cancer cells, which makes folic acid residue a rather good targeting ligand for artificial hybrid nano- and submicron particles (NSPs) [5,6,7,8,9,10]. The application of FA for targeting dates back to the 1940s, i.e., in 1947, Farber and coauthors, based on the earlier studies, used a synthetic derivative of folic acid (4-aminopteroyl-glutamic acid (aminopterin)). This resulted in remissions in children with acute leukemia; therefore, the authors marked pteroyltriglutamic acid and pteroyldiglutamic acid as substances that should be employed in routine cancer therapy [11].

Today, folate-containing NSPs (folate-NSPs) are often used in oncology for chemotherapy [12,13,14,15,16,17,18,19,20,21,22,23,24,25,26], photodynamic therapy [27,28,29], photothermal therapy [21,30], magnetic hyperthermia [31], and a combination of various types of therapy [21,28,31,32,33,34]. A less widespread application compared to chemotherapy is gene delivery, particularly to the central nervous system, due to its ability to cross the blood–brain barrier [35]. Aside from targeted tumor therapy, there are applications such as tumor cell-targeted imaging, magnetic resonance imaging (MRI), and visualization [36,37,38]. Some works are devoted to tumor diagnostics only [39], while others are devoted to theranostics, which is a combination of diagnostics and therapy performed simultaneously [22,40].

Folate has also been used as a targeting agent for noninvasive imaging of atherosclerotic lesions [41], for the targeted delivery of the drug for retinal neovascularization treatment in patients with diabetic retinopathy [42], and in systems for the treatment of numerous inflammatory diseases [38,43,44,45,46,47].

Among different substances that can be components of targeted hybrid NSPs along with folic acid, serum albumins are often proposed, as they possess such necessary properties as biocompatibility, biodegradability, non-immunogenicity, minimal toxicity, good stability, and good reactivity [48,49,50]. Furthermore, albumin has the ability to enhance the tumor targeting of anticancer drugs via interaction with specific receptors overexpressed in several tumor cells. There are multiple examples of drugs that can be bound with albumin-containing NSPs and targeted to cells with FR overexpression, i.e., doxorubicin [13,14,21,26,31,32,33], paclitaxel [12,16,34], docetaxel [20], chrysin [19], baicalin [23], nintedanib [24], gemcitabine [25], etoricoxib [46], vinblastine sulfate [51], methotrexate [52], and polar β-carboline derivatives [27]. Bovine and human albumins are biological macromolecules used in the pharmaceutical industry not only for the delivery of drugs and other substances [53,54,55] but also for wound healing [56], as antioxidants [57,58], and for infusion therapy [59,60].

Focusing on the application of the systems, including FA and albumin (folate-albumin-NSPs), it should be outlined that they are predominantly used for the therapy of cancer [12,13,14,15,16,17,18,19,20,21,22,23,24,25,26,27,30,31,32,33,34,39,46,61], for theranostics [28,62], and in imaging [30,36,38], which is the detection of tumors and the monitoring of cancer treatment. In several cases, the systems containing folate and albumin are used for the treatment of inflammatory diseases (for instance, rheumatoid arthritis [46]). In all the above-mentioned systems, FA residue is either covalently or non-covalently bound to NSPs or albumin.

Although numerous review papers emphasize the applications and structure of FA-functionalized systems, several critical issues remain underexplored. These issues include the coexistence of covalent and non-covalent binding between folic acid and other system components, which leads to the formation of heterogeneous products. Additional overlooked points are the impact of the synthesis solvents/conditions on albumin conformation and the ability of folic acid to decompose under UV light, etc. In most of the articles devoted to folate-albumin conjugates, binding between FA and albumin is confirmed by in vitro experiments with cells and by different physicochemical methods.

In the present review, we focus on the qualitative and quantitative characterization of covalent and non-covalent binding between FA (and its derivatives) and serum albumin. Special attention in this work is paid to the methods confirming binding between the protein and different forms of FA. According to our analysis of the literature, this aspect has not been previously summarized. Since effective biomedical performance, particularly the targeting of the NSPs to tumor or immune cells, is dependent on the successful and stable binding of FA with NSPs, a clear understanding of the binding mechanisms between the FA residue and albumin is of great importance.

The review also analyzes the influence of details of the preparation process (solvents, chemical agents, number of preparation stages, additional conditions) on the binding of FA and albumin. The impact of this aspect is rarely analyzed in the literature, although it can affect the state of folate in the hybrid system, the albumin conformation within the system, and the availability of protein binding sites, which may determine the medical efficacy of the system. Another problem is the absence of, and inconsistency in, quantitative data on FA-albumin ratios in complex nanosystems. We collected and unified the data in one of the manuscript sections.

## 2. Folate Residue and Its Binding in Organisms

**Figure 1:**
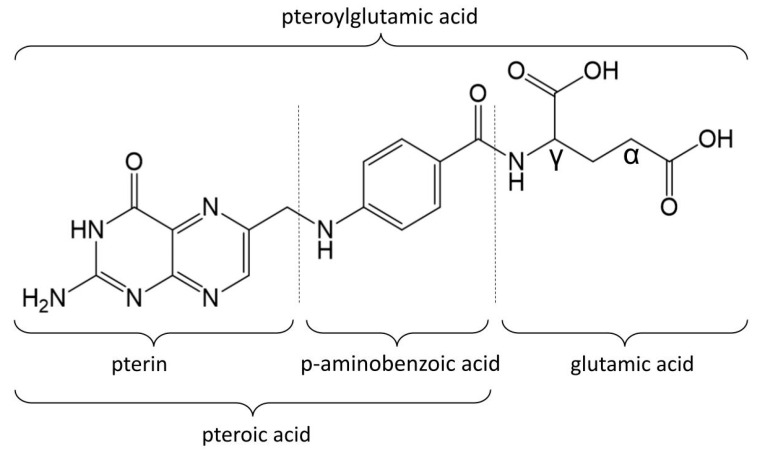
Folic acid molecule.

Folates constitute a group of water-soluble vitamins made up of a pterin ring, a p-aminobenzoic acid, and a γ-linked chain that includes one or more L-glutamic acid molecules, which together form the pteroylglutamic acid core. The structural variations of folates depend on the degree of reduction and substitution on the pterin ring, as well as the length of the glutamate chain. Folic acid is a form of folate consisting of one glutamate residue. In folic acid, a pteridine ring is linked by a methylene bridge to para-aminobenzoic acid and a single glutamic acid molecule (Figure 1). These acids together form the pteroylglutamic acid core of the FA molecule.

Folic acid, as stated, can be used to treat a wide spectrum of diseases. For example, treatment with FA after an acute myocardial infarction for 6 weeks results in improved endothelial function in patients with normo- and hyperhomocysteinemia [1]. Folic acid therapy is also able to significantly delay the progression of chronic kidney disease (CKD) among patients with mild-to-moderate CKD [2]. Folic acid has a promoting effect in the prevention of Alzheimer’s disease, dementia, neuropsychiatric disorders, and cerebral ischemia [3]. As a vitamin, folate is essential during pregnancy for the successful fetal development process and for the prevention of numerous neurological and cardiovascular diseases [4].

Folates found in nature are typically reduced to either dihydro- or tetrahydrofolate and may carry a carbon moiety (i.e., methyl, formyl, methylene, or methenyl). Serum mainly carries 5-methyl-tetrahydrofolate (~80%), which is bound unspecifically, e.g., to albumin with low affinity [63]. Folate has been revealed as a targeting molecule for activated macrophages, which cause or contribute to numerous inflammatory diseases such as rheumatoid arthritis, Crohn’s disease, atherosclerosis, lupus, inflammatory osteoarthritis, diabetes, ischemia reperfusion injury, glomerulonephritis, sarcoidosis, psoriasis, Sjogren’s disease, and vasculitis [38,43,44,45]; thereby, many folate-containing systems have been developed to treat these diseases. There are multiple examples of such applications: a system for noninvasive imaging of atherosclerotic lesions was created [41]; a system for delivery to affected joints during rheumatoid arthritis was developed based on albumin NSPs [46]; Dave and coauthors prepared a system for the targeted delivery of the drug for retinal neovascularization treatment in patients with diabetic retinopathy using gold nanoparticles with polyethylene glycol (PEG) [42]; carbon dots fabricated from FA were shown to effectively delay osteoarthritis pathogenesis [47].

Folates are sensitive to heat, oxygen, and UV light, leading to the formation of inactive products. Due to the hydrophilic properties of folates, they can be easily lost by leaching. It has been demonstrated that folic acid and 5-methyl-tetrahydrofolate undergo glycation in the presence of reducing sugars, particularly fructose [64]. However, numerous methods have been developed to prevent folate degradation, such as the addition of ascorbic acid and polyphenols [64]. Also, the binding of folates with biological macromolecules allows for better preservation of folates. Folates bind to proteins and alter protein structure under irradiation, while proteins improve the photostability of folates.

It was shown that in blood, around 50% of folate is bound to albumin, and the rest is free in solution. The binding of folic acid to albumin (analyzed using [^3^H]folate) is maximal at about pH 6.0 and negligible both above pH 8.0 and below pH 4.5 [65]. Albumin acts as a transporter of folate to tissues, and, due to the low affinity between folate and serum albumin, folate could be displaced from albumin under physiological conditions or in the presence of a substantial number of proteins with a higher affinity to folate, especially folate receptors.

Folate receptors are a group of proteins containing four subtypes (most frequently called isoforms): FRα, FRβ, FRγ, and FRδ. FRα is the most widely expressed receptor isoform with high affinity towards folic acid. FRα is expressed at low levels on the luminal surface of certain epithelial cells in the lung, kidney, choroid plexus, retina, and placenta, and it is often expressed in cancer cells [66], while placenta and hematopoietic cells (such as activated macrophages) express FRβ. FRα and FRβ are membrane-bound proteins anchored to the outer cell surface via a glycosylphosphatidylinositol anchor [67]. The binding of FA to FRα and FRβ is described and shown in [68,69,70]. Pteroic acid is deeply buried in the receptor pocket and stabilized by hydrogen bonds and hydrophobic interactions [68]. On the contrary, the L-glutamic acid of FA remains at the FR pocket entrance, with the α-carboxylic group of glutamic acid involved in binding with FR [71]. In order to preserve the binding affinity between FR and folate, some researchers suggest that conjugation of FA to different agents should be performed through γ-carboxylic acid [72]. Other authors showed that there is no difference between the binding of α- and γ-regioisomers to FR (both in vitro and in vivo) [73], while there is a difference in the distribution of the isomers of conjugates in the organism.

Particularly, it was shown that in the liver, higher nonspecific uptake of the systems with γ-conjugated folate is observed, while in the kidney, nonspecific uptake of the systems with α-conjugated folate is up to two times higher than that of systems with γ-conjugated folate [74]. Therefore, the systems including γ-conjugated folate could not be used to detect liver metastasis.

It is noteworthy that FRs not only participate in receptor-mediated endocytosis of FA but also act as transcription factors and signaling molecules. When FR is cleaved from the glycosylphosphatidylinositol anchor, it can translocate into the nucleus and act as a transcriptional factor. Regulation of gene expression under this transcriptional factor is thought to contribute to oncogenesis [3]. FRs bound with FA were also shown to activate STAT3 signaling, which includes three components: Janus kinase, signal transducer and activator of transcription proteins, and receptors [67]. STAT3 signaling is a proto-oncogene that promotes oncogenic transformation by stimulating downstream angiogenesis and metastasis and either inhibiting or promoting apoptosis. The role of folate and folate receptors in oncogenesis has been actively studied, but with controversial results: some research focuses on the potential of folate to prevent cancer, while other research suggests its contribution to oncogenesis [75,76,77,78].

There is a wide variety of cancer cell lines with overexpressed FR. They include gastrointestinal cancers (colorectal cancer [79,80], pancreatic cancer [81]), cancers of the reproductive system (cervical cancer [82]), adenomas [83,84,85], ovarian cancers [85], testicular cancer [86], breast cancer [43,87,88], lung cancer [89], head and neck squamous cell carcinoma [90], kidney cancer [86], liver cancer [86], nasopharyngeal tumors [86], and other cancer types [67]. For example, an overexpression of FR was shown on the surface of the serous ovarian cancer cell line SKOV-3 [91], cervical cancer cell lines HeLa and SiHa [82], and the colorectal cancer cell line HT-29 [18].

FA (or folate), as a widely used targeting ligand or biovector, can be used as a component of NSPs to target them to various FR-positive cells (FR+), to be taken up by them, and to treat diseases that are associated with FR overexpression, particularly cancer and inflammatory diseases [9,10]. The targeting of drug molecules using NSPs extends the short half-life of the drugs and reduces their toxicity, dose-dependent side effects, and intestinal absorption problems caused by the drugs [52].

## 3. Targeting of Albumin-Containing NSPs

Albumin is present in both blood plasma and interstitial fluid and has many special binding sites capable of transporting various substances, including drugs, which makes it an excellent potential carrier for drugs, particularly to tumor cells [50]. Albumin is known owing to its biocompatibility, biodegradability, non-immunogenicity, minimal toxicity, good stability, and good reactivity [48,49,50]; it demonstrates abilities to reduce thrombogenic activity [92] and the adsorption of blood proteins [93], improve the colloidal stability of NSPs [94,95], prolong their circulation in the blood [96], and regulate the cellular internalization of supramolecular polymer assemblies [97]. Albumin in NSPs (in both albumin NSPs and other variants of albumin-containing NSPs) can also interact with tumor cells since they secrete albumin-binding glycoprotein (secreted protein acidic and rich in cysteine (SPARC)) receptor on the surface, which provides the preferential uptake of albumin NSPs by these cells [48,98].

**Figure 2:**
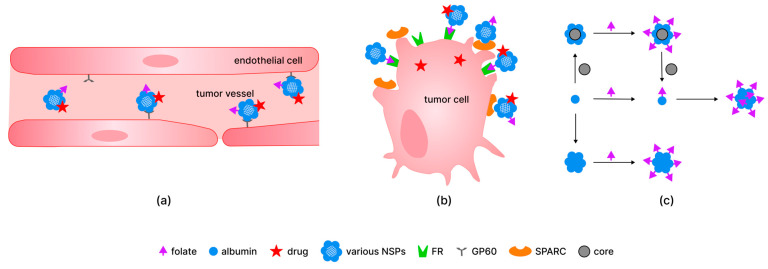
The mechanism of delivery of different systems containing folate residue, albumin, and drug in vessels (**a**) and into tumor cells (**b**); examples of the preparation of the folate-modified albumin-containing systems, particularly based on particles with cores of various (metal, silica, polymer) nature (**c**). Abbreviations: SPARC—secreted protein acidic and rich in cysteine; GP60—glycoprotein 60 receptor; FR—folate receptor.

Regarding the circulation of albumin in organisms, its long half-life (about 12 to 20 days) is maintained by receptor-mediated pathways (mainly by the neonatal fragment crystallizable receptor and, to a lesser degree, by the glycoprotein 60 receptor (GP60), which provide transcytosis of albumin without its degradation by lysosomes [99]). It was also demonstrated that GP60 is present on the surface of cancer cells [100,101]. The combination of albumin and FA residue targeting properties is beneficial for drug transport and diagnostic applications of NSPs (Figure 2).

In addition, albumin, an energy and nutrition resource, can be actively consumed by growing tumors to support the amino acid metabolism of cells. Additionally, individual albumin molecules and albumin in NSPs of particular sizes [102] can accumulate extensively at tumor sites with leaky vasculatures via the effect of enhanced permeability and retention (EPR). Numerous studies show that NSPs with a particle size between 30 and 200 nm can effectively reach the tumor site through the EPR effect [102,103]. However, within this particle size range, the retention and penetration capabilities of NSPs vary considerably. NSPs smaller than 50 nm can penetrate deeply into the tumor regions, but their retention is limited due to cellular efflux and backflow into peripheral blood vessels. For NSPs around 100 nm in size, their relatively large size limits their ability to penetrate deeply into the tumor tissue, but they exhibit strong retention within tumors because they become easily trapped in the extracellular matrix between tumor cells and are less likely to be cleared or eliminated by some way. As noted in [102], nanoparticles in the 100–200 nm range are optimal for maximizing the EPR effect in solid tumors, particularly in organs like the liver and spleen, due to their ability to escape clearance in these organs.

In cells, albumin either remains a part of the NSPs (as was shown using human colon adenocarcinoma (HCT116) and human breast adenocarcinoma (MCF7) cell lines in [104]) or detaches from the surface of NSPs through the reaction with glutathione (since the intracellular level of glutathione is around 5–10 times higher than in blood) [34]. In [34], a targeted nanoplatform was modified by bovine serum albumin (BSA) acting as a biodegradable “gatekeeper,” preventing early drug release and cargo leakage in the blood. Particularly, the internalization of the BSA-modified platform into cells via FRs leads to the detachment of BSA from the system and, therefore, stimuli-responsive (glutathione-initiated) controlled release of drugs in SGC-7901 and MGC80-3 gastric cancer cells in vitro [34].

Other components of folate-functionalized albumin-containing systems could provide additional advantages, particularly for targeting. For example, magnetic cores are not only applicable for magnetic resonance imaging [105] and magnetic hyperthermia (heating under the action of alternating fields) of tumors [31], but they also provide magnetic concentration in tumor tissues.

Certainly, the overall composition of the nanosystem influences targeting as well as further tumor response; for example, drugs or nanozymes, which can be cores in NSPs, are able to influence tumors chemically and also potentiate antitumor immune responses by disturbing tumor metabolism and performing synergistic treatment [106].

## 4. Non-Covalent and Covalent Binding of Folic Acid to Albumin-Containing NSPs

There are multiple medical systems based on FA and serum albumin, i.e., BSA NSPs with folate residue and anticancer substance (fisetin) for tumor-specific targeted chemotherapy of cervical cancer [15]; β-carboline derivatives as photosensitizers covalently linked to folate-tagged albumin for photodynamic therapy (PDT) of human carcinoma [27]; silica NSPs coated with BSA modified with folate and drug (Paclitaxel) and Indocyanine green for combined chemotherapy and PDT of gastric cancer [34]; polymeric micelles functionalized with FA conjugated with BSA and loaded with superparamagnetic iron oxide nanoparticles (SPIONs) for use as a contrast agent for hepatoma detection using targeted MRI [37]; iron oxide NSPs with BSA coating conjugated with FA and labeled with a visualizing agent (fluorescein isothiocyanate) for MRI and intracellular visualization in human brain tumor diagnostics and therapy [38]; etc. Folic acid can be bound to albumin-NSPs at different stages of their engineering (Figure 2c). There are two main approaches to incorporate folate into the structure of complex hybrid systems: (1) FA is bound to individual albumin molecules for subsequent modification; (2) FA is bound to albumin NSPs or albumin-containing hybrid NSPs, etc. As we have shown in more detail before [9], FA can be bound to albumin both at the final step and at the intermediate steps of complex hybrid system preparation.

It has been shown that both systems with covalently and non-covalently bound FA function as targeted NSPs in biological systems. Khalil and colleagues used human serum albumin (HSA) NSPs coated with FA through electrostatic binding for the delivery of the drug doxorubicin to treat renal cell carcinoma [107]. They demonstrated the anticancer efficacy of this system on RCC-GH cell lines. Other authors compared the effectiveness of the non-covalently and covalently bound FA as a targeting agent [61]. It was demonstrated that binding to the cell surface and uptake by the tumor cells increase in the following sequence of samples: non-modified albumin nanoparticles < albumin nanoparticles modified by non-covalently bound FA < FA-conjugated albumin nanoparticles [61]. Also, in [61] it was shown that in the case of covalently bound FA, the uptake of HSA nanoparticles was significantly higher than for systems with non-covalently bound FA.

It is worth noting that covalent binding was likely preferred over non-covalent binding in the majority of works on the creation of hybrid systems for biomedical applications. A fundamental difference between non-covalent and covalent binding is that non-covalent binding is reversible, and the stability of the non-covalent complex depends on external conditions.

By altering the environment of NSPs, the non-covalent binding constant can be increased or decreased, leading to the detachment of FA if necessary. Systems with non-covalently bound FA are shown to be not stable enough in physiological conditions [108] or demonstrate decreased uptake by cancer cells [61]. At the same time, many works are exploring the non-covalent binding of FA to serum albumin [108,109,110,111,112,113,114].

It should be mentioned that covalent binding of folic acid to albumin-containing NSPs can be carried out through various synthetic molecules modifying the surface of hybrid systems, albumin, or FA. The molecules for the modification of albumin-containing NSPs are often polymers based on PEG, which possess numerous benefits. PEG improves the stability of particles in salt solutions [115] and their aggregation resistance. In combination with other polymers PEG provides new drug-binding and release properties to the materials [116,117] and increases their circulation time in the body [118]. Furthermore, the hydrophilic PEG coating on the NSPs prevents nonspecific binding to cells and macromolecules by forming an aqueous layer protecting the surface through the ‘excluded-volume effect’ [119,120,121]. Regarding tumors, it should be noted that PEG, as well as albumin, can increase the accumulation of nanoparticles in the tumor microenvironment as a result of the EPR effect [118]. These factors, in summary, make PEG a promising agent for NSP surface modification for different biomedical purposes. For instance, systems based on albumin and PEG for the targeted delivery of natural substances—namely thymol [118] and naringenin [122]—to cancer cells have been created. Additionally, a PEG linker connected with a folate residue can also be used in the system for early-stage cancer diagnostics, i.e., ovarian cancer, via separating and detecting circulating tumor cells [39]. Despite these promising PEG-containing systems that have demonstrated good performance in a substantial number of applications, some systems containing PEG were shown to be less promising in comparison with the systems containing other linkers: Siwowska and coauthors [123] tested the influence of adding PEG or short-chain alkane linkers to albumin-binding folate conjugates used for radionuclide therapy. It was demonstrated that systems with short-chain alkane linkers showed high tumor uptake (and, therefore, are promising for designing therapeutic systems), while systems with PEG performed with reduced efficiency, probably due to the substantial length of the PEG unit.

**Figure 3:**
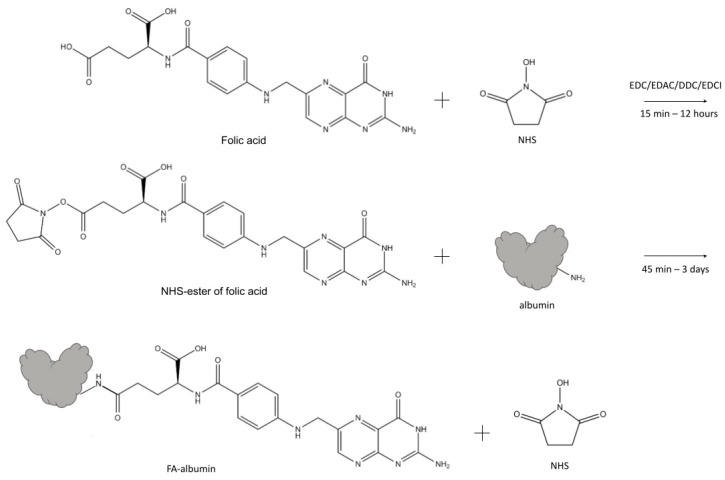
The schematic illustration of one type of synthesis of FA-albumin conjugate: NHS-esters of FA form by EDC/EDAC/DDC/EDCI activation of the terminal carboxylate of FA, followed by the formation of the amide bond with albumin. Abbreviations: NHS—N-hydroxysuccinimide; EDC—N-(3-dimethylaminopropyl)-N-ethylcarbodiimide; DCC—N,N’-dicyclohexylcarbodiimide; EDAC (or EDCI)—1-ethyl-3-(3-dimethylaminopropyl) carbodiimide; FA—folic acid.

Non-covalently bound FA is sometimes used in the systems for medical treatment, but according to some data, the efficiency of these systems could be lower in comparison with that of the systems with conjugated FA. Covalent linking of FA with albumin in most works is performed through carbodiimide chemistry, particularly via NHS-ester of FA or NHS-ester of serum albumin [9]. Different diimide reagents (carbodiimides) are commonly used for the conjugation of FA to albumin [9]. They belong to the group of zero-length cross-linkers and induce the formation of a covalent bond between carboxylates and primary or secondary amines without becoming a part of the final amide-bond crosslink. In some cases, folic acid [14,30,61] was activated by carbodiimides forming O-acylisourea active ester of FA and bound to albumin, while in majority of cases [12,13,14,15,16,17,19,20,22,23,25,26,30,31,34,36,46,51,61,124,125,126] binding between the folate carboxylic group and the amino groups of proteins is obtained via *N*-hydroxysuccinimide (Figure 3). The majority of folate-albumin conjugation nowadays is carried out via this method of linking. When engineering folate-albumin-NSPs for in vivo usage, it is of high importance to avoid toxic cross-linkers and agents, so conjugation of FA to albumin via NHS-ester of FA could be preferable [45] and is often used for chemotherapy [19], photothermal therapy (PTT) [37], combined therapy and PDT [32], combined chemotherapy and magnetic hyperthermia [31], diagnostics [36,38,39], and Rheumatoid arthritis treatment [45], etc.

NHS-ester of FA needs purification by preparative chromatography and further confirmation of the structure. Formation of NHS-ester of FA is usually proved by nuclear magnetic resonance (NMR) [127] or NMR with chromate-mass-spectrometry [128,129], while single UV-Vis spectrophotometry fails to confirm the purity of the product. Additionally, the reaction between NHS-ester of FA and albumin needs relatively mild conditions [130] or physiological pH [131] to perform efficient conjugation. The obtained product (folate-albumin conjugate in its individual form or folate-albumin-NSPs) is rather simple in purification. However, it should be mentioned that in some works, FA is conjugated to serum albumin via N-hydroxysuccinimide ester of serum albumin [27,28].

There is a wide range of conditions suggested for linking between NHS-ester of FA and serum albumin. This bond is thought to form at different pH levels and buffer contents (for example, in Na_2_CO_3_/NaHCO_3_ buffer solution, pH 8.5–10.0 [12,16,17,21,22,24,25,31,36,51,52,126], buffer solutions containing phosphate (PBS with pH 9.1–10.0 [20,23,32], pure phosphate buffer solution with pH 6.3–7.4 [132], and distilled and deionized water [15,26,30,38]). Some of the above conditions may lead not only to covalent binding between FA derivative and albumin but also to NHS-ester hydrolysis [133]. It should be mentioned that the more basic the pH, the more intense the NHS-ester hydrolysis to NHS and FA. According to [131], the half-life of hydrolysis for NHS-ester compounds is 4–5 h at pH 7.0 and 0 °C; this half-life decreases to 10 min at pH 8.6 and 4 °C. Therefore, not only may covalent binding occur between the FA derivative and albumin, but non-covalent binding of FA residues on albumin may also occur, as shown in some studies, which is often not mentioned by researchers. Covalent binding between carboxylic groups of protein and amino groups of FA (and vice versa) can proceed either via ionic interactions or via amidation reactions, depending on pH. The formation of amide is observed at pH 7.5–8.0 (more than 90%) and decreases significantly at pH 11–12. Therefore, high pH conditions should be used with caution, and non-target products of the reaction between the NHS-ester of FA and serum albumin can be expected. Using a medium with neutral, slightly acidic, or slightly basic pH for conjugation allows a product to be obtained that is stable in blood with a pH ~ 7.4. In some works [17,46,126], the NHS-ester of FA is purified and dried for subsequent interaction with serum albumin, while in other papers, the NHS-ester of FA interacts with serum albumin without special purification [19,22,26].

For non-covalent interactions between serum albumin and folic acid, PBS [14], distilled water [30], Tris–HCl (pH 7.4) [109,110], MES buffer [28], and HEPES buffer (pH 7.25) [111] are used. In the reaction mixture, organic solvents (dimethyl sulfoxide (DMSO), ethanol) are frequently present and often come from solutions of FA derivatives (the NHS-ester of [13,15,19,26,30,32,46,51]) and FA activated under carbodiimides [37]. The content of these solvents varied up to 28.6% (*v*/*v*) in the case of DMSO [12] and up to 50% (*v*/*v*) in [18] in the case of ethanol.

At the same time, however, the presence of these solvents in the solution influences the conformation and properties of albumin, predetermining its state and characteristics in the composition of the final NSPs. It was shown that only 2% (*v*/*v*) of the DMSO added to the solution affects the environment of Tyr and Trp residues of albumins: in the case of Tyr, this influence is significant (its fluorescence decreases to negligible values), while for Trp, the effect is weaker and varies depending on the kind of protein (BSA and HSA) [134]. Other research works [135,136] thoroughly studied DMSO-induced transformations of BSA and HSA. Using fluorescence spectroscopy and near-UV circular dichroism [135], as well as Raman and Raman optical activity spectroscopies [136], they showed that at DMSO concentrations up to 10% (*v*/*v*), the native BSA structure was retained [135], while the structure of HSA underwent small changes [136]. According to [135], the bovine albumin starts losing its structure with amounts of DMSO greater than 10% (*v*/*v*) and attains a completely unfolded form in the presence of 40% (*v*/*v*) DMSO. Whereas according to the data of [136], at 50% (*v*/*v*) DMSO in the solution, only partial unfolding of the human albumin is observed, while complete destabilization of the structure takes place at 100% (*v*/*v*) DMSO. This difference can likely be attributed to the methods the authors used: [135] used fluorescence spectroscopy, which is highly sensitive to changes of internal and external factors.

In the case of the other popular organic solvent (ethanol), it was demonstrated that it had toxic effects on BSA, which led to protein denaturation, and the effects increased with the ethanol dose [137]. If the ethanol concentration exceeds 30% (*v*/*v*), the fluorescence peak of albumin (BSA) decreases, and it continues decreasing at 40% (*v*/*v*) of ethanol. A substantial secondary structure disruption is observed: unfolding of the β-turn, β-strand, and α-helix structures of albumin and the formation of intermolecular β-sheet structures [138].

To sum up, quantitative data regarding the specific percentage of organic solvents contained in the solution and significantly affecting protein conformation could vary. However, it can undoubtedly be concluded that an effect of solvents on protein does exist. As stated above, in the range from 2 to 10% (*v*/*v*) DMSO in the solution, the effect is weak and detected only by some methods. When the DMSO percentage is above 10% (*v*/*v*), significant destabilization of the protein structure begins, gradually leading to the completely unfolded form of the protein. In most studies where DMSO is present in the reaction system, its concentration is above 10% (*v*/*v*), and it can be assumed that the protein structure may be partially unfolded under these conditions. Moreover, some conditions may lead to varying extents of folding reversibility. According to our data, none of the authors analyzed their system from the perspective of folding, which may have led to difficulties and irreproducible effects in vitro and in vivo. Also, the reversibility of unfolding should be analyzed.

Different NHS-ester-of-FA)/albumin ratios are used when creating folate-functionalized particles (for example, ~7 in [51] and 143 in [36]), leading to different folate/albumin ratios in the NSPs (see Section 5). The incubation period lasts from 3 min for FA and albumin [111] to 45 min to 3 days [30] for NHS-ester of FA and albumin. In the majority of the analyzed works, the reaction was conducted at room temperature, but sometimes the reaction was carried out under heating (up to 35 °C [34]). It is worth noting that in many research works, this process was performed in darkness [12,13,15,20,21,22,23,26,30,38]. In some cases, the reaction between albumin and NHS-ester of FA was stopped by adding hydroxylamine [61].

It has been shown that when conjugation of FA with macromolecules is performed through a carbodiimide reaction (with and without NHS-ester formation), γ-conjugates are formed as the major product (the selectivity of the reaction ranges from 50 to 90%) [128,129,139]. At the same time, the content of the conjugate after the carbodiimide reaction between FA and serum albumin is rarely analyzed.

**Figure 4:**
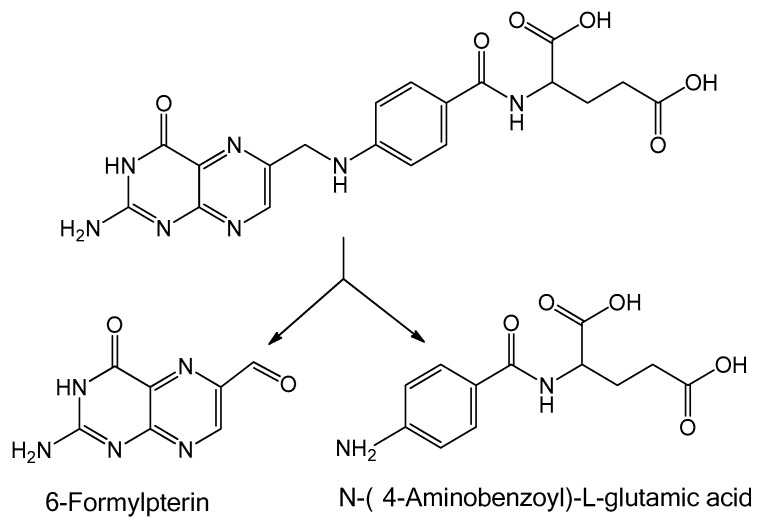
Photodegradation scheme of folic acid.

Therefore, when binding is carried out, some factors should be taken into account. One of these is using conditions mild enough to preserve the structure of the NHS-ester of FA before binding. Another is darkness during the storage of reagents and incubation of the systems, since folic acid is cleaved into p-aminobenzoyl-L-glutamic acid and 6-formyl pterin when exposed to UV irradiation [140] (Figure 4). It should also be noted that binding of FA to proteins (in particular to bovine albumin) partially protects FA from decomposition under UV light [141]; thus, such systems could be used for nutraceutical applications. Another problem is the way FA or FA derivatives are treated (e.g., purification or drying) before interaction with serum albumin. Obviously, these differences in procedures could influence the peculiarities of interaction between serum albumin and FA derivatives and lead to the formation of by-products that sometimes cannot be separated from the main product.

The influence of FA and NHS-ester of FA on serum albumin structure is described in Section 4, as it is important for albumin function.

## 5. Modification of the Structure of Serum Albumin as a Component of Folate-Targeted NSPs

Serum albumin is a water-soluble protein with a well-defined structure that demonstrates extraordinary ligand-binding properties. Albumin is frequently used for the creation of NSPs for biomedical applications [62,142,143]. Therefore, one of the important roles of albumin in folate-targeted albumin-containing NSPs is to provide a platform for binding different drugs and visualizing agents.

Serum albumin was shown to improve the solubility of biologically active substances (i.e., ninthedanib [24]) and participate in the transport of different substances in the body. Its structure is interconnected with its ability for drug transport. In the structure of HSA there are three domains: domain I (residues 1–195), domain II (residues 196–383), and domain III (384–585), which consist of subdomains IA, IB, IIA, IIB, IIIA, and IIIB. HSA possesses two major Sudlow’s sites for the binding of substances. Site I (in subdomain IIA) binds such well-known substances as warfarin, iodipamide, phenylbutazone, azapropazone, and bilirubin; site II (in subdomain IIIA) binds such well-known substances as ibuprofen, tryptophan, and diazepam. The drugs to be delivered by folate-modified NSPs also bind to albumin via Sudlow Site I (chrysin [144,145,146], paclitaxel [147,148], baicalin [149], doxorubicin [150], and methotrexate [151,152]) and Sudlow Site II (chrysin [144,145,146], paclitaxel [147,148], nintedanib [153], gemcitabine [154], and methotrexate [152]). However, biologically active substances are known to be located not only in two Sudlow’s sites, but also in other sites of domains II (polar β-carboline derivatives [155]) and III (gemcitabine [156,157]), as well as in domain I (chrysin [158]) and even the cleft between domains I and III (paclitaxel [147,148] and vinblastine sulfate [159]). BSA has a similar domain structure to HSA. It also should be noted that albumin binding can enhance the delivery and performance of the therapeutic and visualizing compounds (for example, albumin increases the fluorescence of the visualizing agent dimethylindole red [36] and improves the photophysical properties, bioavailability, and photodynamic performance of several tetrapyrrolic photosensitizers [160,161,162,163,164,165], mostly due to preventing their aggregation via complex formation.

Biologically active substances and other ligands are conjugated with albumin nanoparticles via covalent bonding, electrostatic adsorption, or surface coating techniques [48,166]. Targeting ligands, such as antibodies and their fragments and mimetics [167,168], aptamers [169], peptides [170], transferrin and lipoproteins [48] and other low-molecular-weight substances including folic acid [9] can be bonded to NSPs using the advantages of the albumin structure directly, using additional reactive groups (carbodiimide [48,167,170], maleimide chemistry [171], etc.) or through the linker molecules (biotin [20,172,173], PEG [39]). The common approach for FA binding to albumin-NSPs is a carbodiimide chemistry with the subsequent addition of N-hydroxysuccinimide to form an NHS-ester of folic acid. NHS-esters are known to conjugate with the ε-amine in the side chain of lysine or the α-amine of the N-terminus of the protein [15,174,175], and also with various amino acids (serine, tyrosine and threonine, arginine, histidine) [175]. However, the α-amine of the N-terminus of the protein and lysine residues are expected to be the primary reaction sites [175].

There are 59 lysine residues in human serum albumin. Among them, Lys199 is considered the most reactive in the covalent binding of ligands to albumin via NHS-ester. At least 10 other lysines are available on the surface of HSA [176]. The structures of BSA and HSA are 76% homologous [99]. Among the 58 lysine residues in bovine serum albumin, Lys261, Lys350, Lys413, Lys431, Lys471, and Lys474 [177] were shown to interact with the NHS-ester of FA. Acylation of the nitrogen atom of the ε-amino group, release of N-hydroxysuccinimide, and the formation of a stable amide bond occur. Binding of the NHS-ester of folic acid to albumin is a promising way of forming NSPs because binding occurs primarily through the lysine residues of the protein, which minimally affects the protein structure and does not alter the protein conformation.

**Figure 5:**
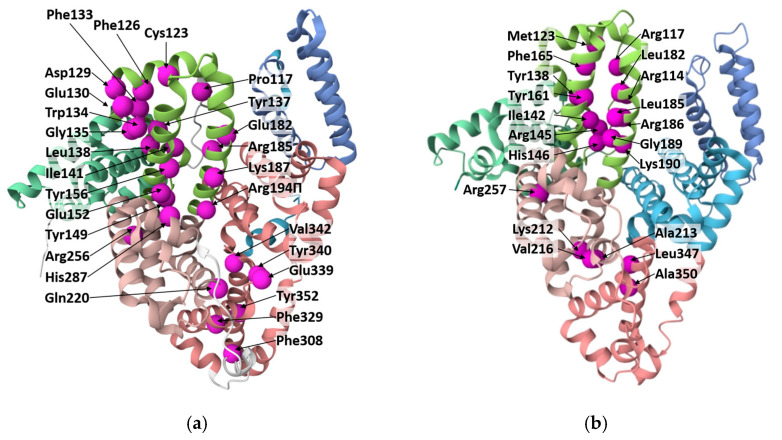
Albumin molecules with amino acids (marked as spheres) involved in binding of FA to BSA (**a**) and HSA (**b**) according to the data obtained by molecular docking and mass-spectrometry. The picture has been drawn with PDB using 4F5S (in the case of BSA) and 6M4R (in the case of HSA).

At the same time, folic acid can interact with albumins in a non-covalent manner (Figure 5). In HSA, folic acid binds within domain I [178] or with subdomain IIA [179,180]. In the first case, it was mentioned by Bourassa and coauthors that Arg114, Arg117, Arg145, Arg186, Gly189, His146, Ile142, Leu182, Leu185, Lys190, Met123, Phe165, Tyr138, and Tyr161 are involved in the binding of FA to glycated and non-glycated HSA [110]. In the second case, for HSA, hydrogen bonds with Lys212, Arg257, and Leu347 and hydrophobic interactions with Val216, Ala350, and Ala213 with folic acid have been discovered [180]. In [113], it was stated that three amino acid residues (Phe309, Phe330, Tyr353) of BSA bind with FA through π-π interactions, and Arg327 binds with FA through hydrophobic interactions. In [15], Tyr149, Tyr340, Glu152, Glu339, Gln220, His289, and Val342 of BSA are predicted to be involved in hydrogen bonds; Tyr156, Lys187, Arg194, and Arg256 are predicted to be involved in hydrophobic and π-π interactions. The other residues of BSA, such as Pro117, Cys123, Phe126, Asp129, Glu130, Phe133, Trp134, Gly135, Tyr137, Leu138, Ile141, Glu182, and Arg185 may also be involved in coordination.

It is noteworthy that there are no amino acid residues involved in the binding of both FA forms (initial FA and NHS-ester of FA), which could indicate that the mechanism of the reaction alters significantly depending on the FA form. In the case of binding between NHS-ester of FA and albumin, it is shown that the major pathway of interaction focuses on the lysine residues and their environment [175], while modification of lysine residues does not cause protein denaturation [130,178], which can be considered an advantage of FA conjugation to serum albumin via NHS-ester form.

After the addition of FA, the content of α-helix in HSA is demonstrated to decrease, while the content of other structures (β-sheet, β-turn structure, and random coil) increases. Especially at high FA concentrations, the addition of FA can affect the protein conformation by partially unfolding it [112]. Besides, the FA binding site is identified within residues 117–185 for BSA and within residues 117–190 for HSA. Rearrangement of the secondary structure of both HSA and BSA could take place due to binding [109].

Therefore, the conjugation of NHS-esters of FA to albumin can be considered the most common and reliable way of folate binding compared to non-covalent binding between FA and albumin. Nevertheless, it is not clear in many works which form of the NHS-ester of FA and which grade of its purification are used for conjugation.

As we stated above (see Section 4), protein conformation could change not only because of folate binding but also because of organic solvents. For instance, the presence of a small amount of organic solvents during the synthesis of the system can lead to changes in the binding constants of different substances and albumin. It was demonstrated that the addition of DMSO at a concentration of 5–10% (mol/mol) increases the constants of curcumin binding to HSA [181]. In the case of curcumin [181], it was proposed that this effect is probably related to the better solvation of the drug and, especially, its associates in the solution in the presence of DMSO < 10% (mol/mol), whereas higher concentrations of DMSO > 10% (mol/mol) result in decreased binding constants due to the competing processes between the protein and the non-aqueous solvent for curcumin [181]. DMSO at a concentration up to 10% (*v*/*v*) is shown to increase isoniazid (an anti-tuberculous drug) binding to BSA [182]. For DMSO content > 10% (*v*/*v*), the binding constant declines owing to the competitive process between DMSO and isoniazid for binding with albumin [182]. Thus, the presence of different substances in the mixture of FA derivatives and albumin should be taken into account since it affects the functioning of the protein on its own and in the composition of NSPs.

Protein conformation changes due to FA derivative binding could significantly affect protein functioning. We expect that it could also influence drug transport and drug release.

## 6. Confirmation and Quantification of Covalent Binding Between Folic Acid and Albumin

When studying the interactions of different forms of FA (initial FA, activated FA, NHS-ester of FA) with albumin (BSA or HSA) in the various forms shown in Figure 4 (individual albumin; albumin in the form of protein NSPs; albumin on the surface of organic, inorganic, or hybrid NSPs), different physicochemical methods can be applied.

Often, it is UV-Vis spectroscopy [12,13,14,15,16,20,23,25,28,30,33,36,45,51,52,183]. In a smaller number of cases, Fourier transform infrared spectroscopy (FTIR) [13,15,19,22,23,26,30,32,39,46,183], methods of size exclusion chromatography (SEC) [61], high-performance liquid chromatography (HPLC) [18], and gel-filtration chromatography [12,26] followed by UV-Vis spectroscopy, differential scanning calorimetry (DSC) [15,19], and ultraviolet matrix-assisted laser desorption/ionization time-of-flight mass spectrometry (UV-MALDI-TOF MS) [27] are used. Quantification via additional chemicals can also be carried out. For example, in [61], the availability of the amino groups of albumin that changed due to folate binding was estimated by UV-Vis spectroscopy. In [24,184], particular amino acids were studied using fluorescence measurements. Biological confirmation of FA-albumin conjugation (particularly without physicochemical confirmation) in NSPs is also used. Different approaches to folic acid-albumin binding confirmation and quantification (particularly those making possible the calculation of folate/albumin ratio in folate-targeted albumin-containing NSPs) are listed in Table 1.

It is worth noting that researchers use UV-Vis spectroscopy to investigate systems containing FA in various ways. Xu and coauthors [36] applied this method to show that the spectrum of folate-albumin-NSPs with dimethylindole red (Dir) possesses characteristic peaks of both folate and Dir. Some authors [13,15,20] have investigated systems including conjugated FA and calculated the amount of bound folate based on characteristic absorption peaks using an FA calibration curve. Other authors, on the contrary, have analyzed the amount of unconjugated folate in the reaction mixture. Du and coauthors [26] separated unreacted FA from the reaction mixture by chromatography and then calculated the binding ratio of FA to albumin using the regression equation of a standard curve obtained with different concentrations of FA (the so-called recovery method). Another example is the research of Shen and coauthors [14], where unconjugated FA was separated by centrifugation, followed by calculating the amount of FA in supernatants on the basis of an FA calibration curve. An approach involving tryptic hydrolysis was used in [16,21,23,25,51,126]: the amount of folate released after hydrolysis was calculated using a calibration curve [25,126] or in relation to the NHS-ester of FA reference [16,51].

This method is used in the majority of studies, either by cleaving the system via tryptic hydrolysis or by assessing the amount of FA directly in the conjugate using calibration curves. These approaches have certain advantages due to more accurate quantitative assessment, but they do not evaluate the state of the FA residue within the conjugate. As mentioned earlier, FA is sensitive to many parameters (medium composition, light, etc.), and it is possible that FA residue in a partially degraded state could remain linked to NSPs.

FTIR can also provide comprehensive information on the changes in the functional groups of the final mixture, particularly in complex systems, compared to the primary substances. Among the analyzed works, FTIR is frequently used to characterize binding between FA and albumin [13,19,22,26,39,46]. All of the researchers showed the presence of FA functional groups in the synthesized systems. Some authors also showed the appearance of a new amide bond between FA and albumin [13,19,22].

Using DSC, Nosrati and colleagues demonstrated the formation of chrysin-BSA-FA NSPs and the absence of any unconjugated folic acid in the product [19].

Ulbrich and coauthors compared the conjugation of carbodiimide-activated FA to HSA nanoparticles with FA non-covalently bound to HSA nanoparticles using SEC [61]. It was shown that the amount of FA bound to HSA was lower in the case of conjugation than in non-covalent binding (7.4 µg folate per mg HSA-NSPs (albumin NSPs) for covalent binding and about 10.22 µg folate bound per mg albumin NSPs). Using o-phthalaldehyde, they showed that the quantity of amino groups on the HSA-NSPs surface decreased after the reaction with FA; the extent of this decline correlated with the amount of FA bound to HSA-NSPs, thus confirming that covalent binding of FA to HSA amino groups took place. Ulbrich and colleagues [61] tested the particle stability of albumin NSPs with covalently and non-covalently bound FA in the cell-culture medium via particle size measurements and also performed cell viability and uptake assays. Comparing conjugated and non-covalently bound FA, the authors stated that FA non-covalently bound to albumin NSPs increased albumin NSPs binding to cancer cells to a much lesser extent than folate conjugation to HSA NSPs. Intracellular uptake of albumin NSPs with conjugated FA was significantly higher than with non-covalently bound FA.

Table 1 illustrates that UV-Vis spectroscopy allows for obtaining quantitative characteristics of FA conjugation to individual serum albumin and to serum albumin in hybrid or protein NSPs via the NHS-ester of FA or via other mechanisms of binding. These characteristics are often expressed as mass and/or molar ratios. When FA is bound to individual albumin, the binding ratio ranges from 9.5 to 40.0 moles of folate per one mol of albumin [12,13,15,20,26], while the same characteristics for FA binding to NSPs range from 0.3 to more than 33.6 moles of folate per mol of albumin [14,18,61]. It is likely that the reduced values of FA binding to NSPs are due to the reduced access of FA to the binding sites of albumin. The synthesized particles have varying amounts of folic acid residues on their surfaces and different ratios of folate to albumin residues, which may also be related to NSPs’ effectiveness and determine their capacity for binding, transport, and drug delivery. Furthermore, a high folate-to-albumin ratio might be associated with the presence of both non-covalent and covalent binding, since these types of binding are in different binding regions of albumin (see Section 5). This phenomenon receives little attention in the literature.

It is worth noting that in some studies [12,14,26,61] the amount of folic acid bound to NSPs is calculated by measuring the amount of unbound FA and subsequently subtracting the detected FA from the total amount of added FA (or FA derivative). Folate-containing NSPs for biomedical applications typically undergo numerous stages during their preparation. At each step of system preparation and FA isolation for quantification, losses of FA concentration could occur due to the physical and chemical impact of various agents. These losses are often not taken into account when folate is estimated. Such an error can be avoided by directly measuring the amount of FA in the conjugate composition, as was done in the majority of the analyzed papers.

Focusing on the physical parameters, the size and morphology of the obtained particles are often estimated using transmission electron microscopy, dynamic light scattering, scanning electron microscopy, laser particle size analysis, and atomic force microscopy [185]. Additionally, zeta potential, which is a fundamental parameter for predicting the stability of colloidal systems, is sometimes measured [13,16,22,46].

Of specific interest is the work of Bilthariya and coauthors [46], who studied the stability of their system upon storage under the influence of a variety of environmental factors, such as temperature, humidity, and light, by evaluating the particle size and drug (etoricoxib) content in NSPs after 60 days of storage. They demonstrated that more than 85% of etoricoxib remains bound to the NSPs after 60 days of storage at different temperatures (4 ± 2 °C and 37 ± 2 °C), which indicates that drug encapsulation is stable enough. Although drug encapsulation into NSPs and its release are frequently analyzed by various research groups, studies estimating the retention of folate availability in the system during storage and under the influence of various physical factors are rather scarce. This oversight may significantly affect the overall effectiveness of such biomedical systems, given the crucial targeting properties of folate.

**Figure 6:**
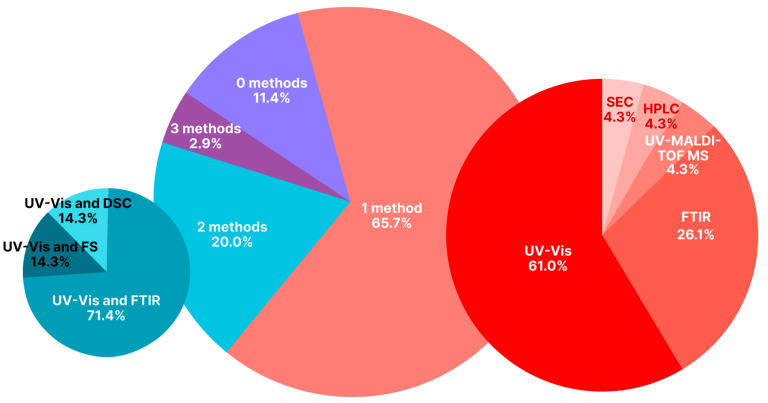
Distribution of physicochemical methods used to confirm the binding of FA with albumin-containing NSPs (particularly albumin NSPs) in the papers devoted to folate-albumin conjugates and presented in Table 1. List of abbreviations: UV-Vis—UV-Vis spectroscopy; FS—fluorescence spectroscopy.

To sum up, UV spectroscopy and/or FTIR are frequently used. Sometimes DSC, X-ray diffraction, UV-MALDI-TOF MS, HPLC, and SEC are used. It is worth noting that often (in only around 1/4 of reviewed cases; see Table 1 and Figure 6) 2–3 physicochemical methods are used simultaneously for assessing the binding. At the same time, in around half of the analyzed articles, the binding was characterized quantitatively.

To summarize the previously mentioned data on hybrid NSPs with FA bound to albumin via a carbodiimide reaction, such multicomponent hybrid systems are characterized by the following physical parameters: NSP particle size and shape, morphology of the NSP surface, and zeta potential of NSPs. The presence of therapeutic and visualizing agents, the presence of folate and its content, as well as the effectiveness of drug encapsulation in the NSPs composition, are estimated. However, there is a lack of articles describing such quantitative characteristics as the binding ratio for amide bond formation and the ratio of non-covalent or covalent binding between the folate residue and albumin in NSPs used for specific biomedical tasks. A set of procedures (for example, washing the resulting systems) performed by researchers and followed by UV-Vis spectrophotometry is commonly considered sufficient to state that the expected type of bond is formed. We should emphasize that covalent binding requires additional methods for confirmation, and single UV-Vis spectrophotometry is sufficient only when evidence has already been obtained simultaneously by UV-Vis spectrophotometry and other methods under the same conditions of binding. Overall, most studies apply only a few methods for characterizing binding, resulting in many developed systems remaining poorly characterized. That is why a lack of data regarding complex composition occurs.

In vitro and in vivo conditions are also used to determine how effective folate is as a targeting moiety in such NSPs. The methods of confirming the binding of the FA residue to the NSPs, based on the efficiency of internalization into cells and/or the therapeutic effect of folate-albumin particles compared to a control on cells or living organisms, for example, without the FA residue, also do not distinguish between non-covalent and covalent binding. It has been shown that systems with both covalently and non-covalently attached folate are effective for targeting. When only one type of system is analyzed in a particular study, the presence of a biological effect alone cannot demonstrate that the binding occurred covalently.

Additionally, there is a lack of information about the stability of these systems under different conditions and during storage, which is especially important for determining the expiration date of NSPs with biomedical applications. Studies emphasizing covalent binding of the folic acid residue as a targeting ligand constitute the majority of the analyzed literature. We assume the following situation occurs: while the biological effect is studied for systems of complex composition, detailed physicochemical analysis can mainly be carried out for very simple systems, such as the folic acid molecule and albumin.

## 7. Confirmation and Quantification of Non-Covalent Binding Between Folic Acid and Albumin

Non-covalent binding of FA to albumin is characterized rather thoroughly via thermodynamic parameters of the interaction between albumin and FA (enthalpy change, entropy change, and free energy change) determined by DSC [108,111,112] or isothermal titration calorimetry [113]; apparent binding constants obtained through cyclic voltammetry [111,112]); adsorption constant of FA at HSA-modified Au electrode surfaces measured by electrochemical impedance measurements [111,112]; and constants of binding obtained by fluorescence measurements [109,110,111,112,113,114,161,166,180,186]. Binding constants for albumin and folic acid are given in Table 2 and were obtained by fluorescence spectroscopy, predominantly from the protein fluorescence. It should be noted that albumin fluorescence is mostly related to the tryptophan residue. HSA includes one tryptophan (Trp214) among 585 amino acid residues, while BSA includes two tryptophans (Trp134 and Trp212) among 583 amino acid residues.

Fluorescence spectroscopy provides an effective estimation of binding between FA and albumin. This method does not require additional sample manipulation. In contrast, data obtained using electrochemical measurements and surface plasmon resonance (SPR) methods cannot be directly extrapolated to FA–albumin binding in solution because immobilization of albumin enhances its affinity for FA compared to the free protein [111]. It was shown that estimating the binding constant using electrochemical measurements can give values five times higher than those obtained by fluorescence spectroscopy [111]. This increase is due to the changes in the orientation of albumin binding sites towards free FA caused by the confinement of albumin on the gold surface, which leads to the formation of a stronger complex in the case of electrochemical measurements compared to fluorescence quenching. For SPR measurements, the binding constants are more than 200 times higher than those obtained from the fluorescence data. This large difference could be caused by the different immobilization methods: the electrochemical method uses drop casting, while SPR uses flow adsorption. Researchers suggest that multilayer BSA film formation in the flow used in SPR facilitates the ability of FA molecules to find albumin binding sites [111].

As shown in the above-cited papers, the dynamic quenching constant (K_SV_) varies in the range of (1.55–10.00)*10^4^ L*mol^−1^ for BSA and (0.282–8.97)*10^4^ L*mol^−1^ for HSA, and the bimolecular quenching rate constant (K_q_) varies in the range of (8.94–100.00)*10^12^ L*mol^−1^*s^−1^ for BSA and in the range of (0.408–13.50)*10^12^ L*mol^−1^*s^−1^ for HSA. The quenching constant of FA binding to albumin is significantly higher than the Kq associated with collisional scattering processes. This indicates that the quenching mechanism is not due to dynamic collisions but rather results from complex formation, which is characteristic of static quenching [114]. The observation that temperature changes do not substantially affect the constants [111] also confirms complex formation between FA and albumin. According to the authors’ suggestions [111] regarding BSA, the binding process between FA and serum albumin is a moderate interaction in which one molecule of FA binds to one molecule of albumin: this interaction is a spontaneous exothermic process driven by electrostatic forces, van der Waals interactions, and hydrophobic forces.

Isothermal titration calorimetry revealed that the complexation of FA to albumin is a two-step binding process without intermediates, and that binding of folic acid to BSA does not significantly alter the protein conformation [113].

Liwinska and coauthors also measured the zeta potential and showed that under physiological and experimental conditions (pH 7.40, T 310 K (36.85 °C)), the complex of HSA and FA is unstable [108]. On the one hand, the authors [108] suggest that this instability, when FA is used as a therapeutic component of biomedical systems, has a promising effect due to the fact that only the unbound part of FA has a therapeutic effect. In contrast, the application of FA as a targeting substance is limited owing to the lack of stability of binding between the protein and FA. This is likely a reason why the systems with non-covalently bound FA demonstrated low performance after being added to the cell culture medium [61].

Therefore, there are many methods that can be used to confirm and study non-covalent binding between folic acid and serum albumin. Among them, some methods allow obtaining quantitative information that could differ from one method to another due to the differences in the performance of the method. The majority of quantitative information is obtained using fluorescent measurements. This method does not require special sample preparation, so extrapolation of the results obtained by the method to in vivo applications and engineering is easier than for other methods.

It is worth noting that qualitative and quantitative assessment of binding allows us to predict the behavior of systems in living organisms (how stable the system will be when introduced in vivo, what modifications can occur with the system in vivo (or in cells), and in what form it will presumably be excreted from the body), as well as the stability of these systems during storage and under various physical factors.

## 8. Conclusions

Today, folic acid is used as a part of hybrid nanosystems containing natural and synthetic macromolecules. In all these systems, using FA (or folate) as a targeting agent allows for the enhancement of system targeting. There are a wide variety of systems, including folic acid and albumin, that are used for medical purposes, particularly for disease treatment and diagnostics. The prevailing number of studies developing folate-albumin systems are aimed at therapy through chemical substances and/or physical impacts (i.e., chemotherapy, PDT, PTT, and magnetic hyperthermia).

The design and engineering of NSPs aimed at biomedical applications often integrate the folate residue to NSPs through conjugation. The popular approach of folate-albumin conjugation is carbodiimide chemistry which is sometimes supplemented with addition of NHS, in which folic acid is converted into O-acylisourea or NHS-ester forms and added to albumin or albumin-containing NSPs in such a form. The probable main reason for the prevalence of this binding approach is the relatively higher stability of the albumin-folate conjugate obtained via the NHS-ester form, compared to FA coordination. But even when folic acid is converted into the ester forms, binding can occur not only predominantly via a covalent mechanism but also by a non-covalent mechanism, leading to heterogeneity of the resulting product. We suppose that in some cases there are both covalent and non-covalent bindings of various FA derivatives with serum albumin. Since there are few comparisons between the forms of binding, it cannot be conclusively stated that non-covalent binding is unsuitable for binding folic acid as a targeting agent. The use of non-covalently bound FA in hybrid systems for biomedical purposes is very limited. We expect that this limitation may be due to the lower efficiency in binding to cancer cells and in cellular uptake and due to the above-mentioned relatively low stability of binding of FA compared to the NHS-ester of FA with NSPs.

It is worth noting that the amount of reliable data related to the binding properties of FA covalently bound to albumin is limited. The characteristics obtained for this binding vary significantly and, moreover, were controversial in some cases. At the same time, binding of FA with albumin through non-covalent binding has been characterized with substantially more detail than the conjugated form of FA.

Also, it should be noted that there are difficulties in working with folic acid and its derivatives that often remain beyond the scope of articles or the attention of researchers. Specifically, (1) the chemical structure of folic acid is photosensitive, (2) complexes may degrade depending on the conditions of the binding reaction, and (3) the purity of the NHS-ester in the synthesis is not evaluated, although it is known that the reaction can be accompanied by a large number of by-products formed at different stages.

An aspect that researchers do not address in their works is the effect of the system preparation process on the conformation and performance of albumin within the system. It was shown that chemical agents and solvents used during system preparation and afterward could lead to alterations in the protein’s secondary and tertiary structure and impact the functioning of the protein itself and within NSPs.

## Figures and Tables

**Table 1:** Qualitative and quantitative confirmation of FA covalent binding with different systems: serum albumin or albumin-containing NSPs.

System Composition	System Preparation				Physicochemical Confirmation of FA Binding to Systems				Testing Biological Effects of Systems			Reference
	Form of FA in Which it is Used for Binding	Form of Albumin to Which FA is Bound	Conditions of Binding of FA or FA Derivate to Albumin	Additional Components of the System	Approach Used with UV-Vis Spectroscopy to Identify Presence of Folate in System and Quantify Folate in the Final System	Amount of Folate Conjugated to Albumin, mol of Folate Per mol of Protein	FTIR	Other Methods (Accompanying UV-Vis and FTIR or Instead of Them)	With and Without Folate on Cells	with Folate on FR+ and FR− Cells (or FR Normal Cells)	With and Without Folate on Mice	
folate-(BSA NSPs)-dimethylindole red	NHS-ester of FA	BSA NSPs with dimethylindole red	saturated NaHCO_3_; incubation for 24 h	-	Presence of folate was identified by characteristic peak of FA	-	-	-	-	+	-	[36]
folate-(BSA-containing NSPs)	FA-PEG_2K_-NHS	BSA-containing magnetic NSPs	phosphate buffer (0.01 M, pH 7.4); incubation for 3 h	-	-	-	+	-	-	+	-	[39]
folate-(HSA-containing NSPs)-(5-fluorouracil)-Curcumin	NHS-ester of FA	HSA-containing magnetic NSPs	carbonate/bicarbonate buffer (0.05 M, pH 9.8); incubation for 12 h; darkness	(5-fluorouracil), Curcumin	-	-	+	-	+	-	-	[22]
folate-(BSA NSPs)-Chrysin	NHS-ester of FA	BSA NSPs with Chrysin	water and DMSO with pH adjusted to 8.5; overnight incubation	-	-	-	+	Differential Scanning Calorimetry	+	-	-	[19]
folate-(HSA NSPs)-Cabazitaxel	NHS-ester of FA	HSA NSPs with Cabazitaxel	carbonate/bicarbonate buffer (0.2 M, pH 10.0); incubation for 2 h		The system was subjected to tryptic hydrolysis; the amount of folate in cleaved system was determined using calibration curve of NHS-ester of FA (cleaved HSA NSPs-Cabazitaxel used as blank control).	>1.8:1 (11.96 ± 3.11 μg FA per 1 mg of folate-(HSA NSPs)-Cabazitaxel)	-	-	+	+	-	[126]
folate-(BSA-containing NSPs)-doxorubicin	NHS-ester of FA	BSA	water and DMSO with pH adjusted to 10.0 using 0.2 M NaOH; incubation for 24 h; darkness	graphene oxide-based NSPs, doxorubicin	Content of folate was calculated using calibration curve	19.6:1 (126.5 µg FA per 1 mg of BSA)	+	-	+	+	+	[13]
folate-(BSA-modified micelles containing SPIONs)	FA activated under action of carbodiimide reagent (EDC)	BSA	water and DMSO, incubation for 4 h; darkness	polymeric micelles containing superparamagnetic iron oxide nanoparticles (SPIONs)	-	-	+	-	+	+	-	[37]
folate-(BSA NSPs)-Etoricoxib	NHS-ester of FA	BSA NSPs with Etoricoxib	phosphate buffer (pH 7.4) and DMSO with pH adjusted to 10.0 using 1 M carbonate/bicarbonate buffer solution, incubation for 45 min	-	-	-	+	-	-	-	+	[46]
folate-HSA-docetaxel obtained from docetaxel-HSA and folate-HSA	NHS-ester of FA	HSA	phosphate buffer (0.1 M, pH 9.1); overnight incubation; darkness	docetaxel-HSA	Content of folate in conjugate was calculated using calibration curve	4–6:1 (for 18 µmol FA); 30–40:1 (for 0.3 mmol FA)	-	-	+ (only with folate)	-	+ (only with folate)	[20]
(folate-BSA-containing NSPs)-paclitaxel	NHS-ester of FA	BSA	carbonate/bicarbonate buffer (pH 8.5, 0.2 M); incubation for 10 h; darkness	lipoprotein-mimic nanocarrier, paclitaxel	The unreacted folate was separated from the reaction mixture by gel filtration on a Sephadex G-25 column and calculated using calibration curve for FA	(12.7 ± 0.3):1	-	-	-	-	+	[12]
folate-BSA-doxorubicin	NHS-ester of FA	BSA	water and DMSO; incubation for 10 h; darkness	doxorubicin	The unreacted folate was separated from the reaction mixture by gel filtration; the ratio of FA binding to BSA were determined by the recovery method (the regression equations of standard curves were obtained by different concentrations of FA)	(9.5 ± 0.3):1	+	-	+	-	-	[26]
folate-(BSA NSPs)-doxorubicin	FA activated under action of carbodiimide reagent (EDAC)	BSA NSPs with and without doxorubicin	phosphate buffer; incubation varies from 4 to 24 h	doxorubicin	Content of folate was calculated using calibration curve in the supernatants of the solutions after conjugation	from >0.4 to >1.7:1 for folate-BSA NSPs from >0.3 to >1.6:1 for folate-(BSA NSPs)-doxorubicin (6.42–26.9 nmol FA per 1 mg of folate-BSA NSPs for 4–24 h of conjugation 3.94, 20.9 nmol per 1 mg of folate-(BSA NSPs)-doxorubicin for 4 and 16 h of conjugation 1.1 − 4.6 × 10^4^ mol FA per mol of folate-BSA NSPs 1.6 − 8.2 × 10^4^ mol FA per mol of folate-(BSA NSPs)-doxorubicin)	-	-	+	-	-	[14]
folate-(HSA NSPs)	FA activated under action of carbodiimide reagent (EDC)	HSA NSPs	0.1 N NaOH; incubation for 1 h; reaction was stopped by adding hydroxylamine	-	SEC with calculation of unbound FA by characteristic peak of FA	>1.1:1 (7.40 ± 0.90 µg FA per 1 mg of HSA NSPs; the percentage of binding to the surface of the nanoparticles amounted to 95.8 ± 0.4% of the added folic acid)	-	-	+	+	-	[61]
folate-(BSA NSPs)-paclitaxel	NHS-ester of FA	BSA NSPs with paclitaxel	carbonate/bicarbonate buffer (0.1 M, pH 10.0); incubation for 12 h	-	The system was subjected to tryptic hydrolysis; the amount of folate in cleaved system was determined using NHS-ester of FA reference (cleaved BSA NSPs with paclitaxel used as blank control)	1.1:1 (9.22 μg FA per 1 mg of BSA NSPs)	-	-	+	-	-	[16]
folate-(BSA NSPs)-vinblastine sulfate	NHS-ester of FA	BSA NSPs	water and DMSO with pH adjusted to 10.0 using 1 M carbonate/bicarbonate buffer; incubation for 45 min	vinblastine sulfate	The system was subjected to tryptic hydrolysis; the amount of folate in cleaved system was determined using NHS-ester of FA reference (cleaved BSA NSPs used as blank control)	47.4:1 (383.996 µM FA per 1 g of BSA NSPs)	-	-	-	-	-	[51]
(folate-BSA NSPs)-fisetin	NHS-ester of FA	BSA	water and ethanol; incubation for 12 h; darkness	fisetin	Content of folate was calculated in conjugate using calibration curve of FA	14.2:1 (94.64 ± 1.07 % of FA was conjugated with BSA)	+	Differential Scanning Calorimetry, X-ray diffraction	+	-	-	[15]
folate-(HSA-containing NSPs)-Curcumin-(5-fluorouracil)	FA activated under action of carbodiimide reagent (EDC)	HSA-graphene oxide NSPs with Curcumin and 5-fluorouracil activated under action of carbodiimide reagent (EDC)	water and ethanol; incubation for 4 h at 4 °C	-	Amount of free FA in the system was calculated via HPLC	>33.6:1 for folate-(HSA-containing NSPs); >32.9:1 for folate-(HSA-containing NSPs)-(5-fluorouracil); >33.2:1 for folate-(HSA-containing NSPs)-Curcumin-(5-fluorouracil) (FA has 16.25 ± 1.04% of the total weight of folate-(HSA-containing NSPs)-Curcumin-(5-fluorouracil); 17.05 ± 1.16% of the total weight of folate-(HSA-containing NSPs)-(5-fluorouracil); 18.16 ± 1.05% of the total weight of folate-(HSA-containing NSPs)	-	-	+	+	-	[18]
folate-(BSA NSPs)-gemcitabine	NHS-ester of FA	BSA NSPs with gemcitabine	carbonate/bicarbonate buffer (0.2 M, pH 10); incubation for 1 h	-	The system was subjected to tryptic hydrolysis; the amount of folate in cleaved system was determined using NHS-ester of FA reference (cleaved BSA NSPs with gemcitabine used as blank control)	>1.3:1 (8.49 ± 2.51 µg FA per 1 mg of folate-(BSA NSPs)-gemcitabine)	-	-	+	+	-	[25]
folate-(BSA NSPs)-doxorubicin-(IR-780)	NHS-ester of FA	BSA NSPs with doxorubicin and IR-780	water and DMSO, pH adjusted to 9–10 using carbonate/bicarbonate buffer (0.4 M); incubation for 12 h, darkness	-	Presence of folate was identified by characteristic peak of FA in folate-(BSA NSPs)-doxorubicin-(IR-780)	-	-	-	-	-	-	[21]
folate-(BSA-containing NSPs)-chlorin e6	FA	BSA and Mn:CuSe containing NSPs mixed with carbodiimide reagent (EDC) and Sulfo-NHS	MES buffer; incubation for 2 h	chlorin e6	Presence of folate was identified by characteristic peak of FA in folate-(BSA-containing NSPs)-chlorin e6	-	-	-	-	+	-	[28]
(folate-BSA-containing NSPs)-Indocyanine green-paclitaxel	NHS-ester of FA	BSA	DMSO; incubation for 24 h at 35°C	mesoporous silica NSPs, paclitaxel	Presence of folate was identified by characteristic peak of FA in folate-BSA	-	+	Dynamic light scattering	+	+	-	[34]
folate-(BSA NSPs)-methotrexate	NHS-ester of FA	BSA NSPs with methotrexate	water and DMSO, pH adjusted to 9–10 using carbonate/bicarbonate buffer (1.0 M); incubation for 12 h	-	Presence of folate was identified by characteristic peak of FA in folate-(BSA NSPs)-methotrexate	-	-	-	+	-	-	[52]
(folate-BSA NSPs)-baicalin	NHS-ester of FA	BSA	phosphate buffer (1.5 M, pH 10.0); overnight incubation; darkness	baicalin	The system was subjected to tryptic hydrolysis; the amount of folate in cleaved system was evaluated using calibration curve of NHS-ester of FA	9.9:1 (66.45 ± 1.78 μg FA per 1 mg of BSA)	+	-	+	-	-	[23]
folate-(BSA NSPs)-doxorubicin-chlorin e6	NHS-ester of FA	BSA NSPs with chlorin e6 and doxorubicin	ethanol and phosphate buffer; overnight incubation	-	-	-	+	-	-	+	-	[32]
folate-(HSA-NSPs)-(gold nanorods)-doxorubicin	FA activated under action of carbodiimide reagent (EDC)	HSA NSPs	NaOH and water, incubation for 1 h	gold nanorods, doxorubicin	Presence of folate was identified by characteristic peak of FA in FA-BSA; the unreacted FA in the supernatant was quantified	Percent of HSA NSPs conjugated with folate from total amount of HSA NSPs was 64.6%	-	-	+	-	-	[33]
folate-BSA-gold nanostars	NHS-ester of FA	BSA	DMSO and water; incubation for 3 days; darkness	gold nanostars	Presence of folate was identified by characteristic peak of FA in FA-BSA; the number of folate molecules linked to each BSA was calculated using calibration curve of FA	7.5:1	+	-	+	-	-	[30]
folate-BSA-β-carbolinium conjugate	FA	BSA linked to β-carbolinium in the presence of carbodiimide reagent (EDC) and NHS	water; incubation for 24 h	-	Amount of folate was quantified via Ultraviolet matrix-assisted laser desorption/ionization time-of-flight mass spectrometry (UV-MALDI-TOF MS) analysis	7–10 molecules of FA per BSA-β-carbolinium conjugate	-	-	+	-	-	[27]
(folate-BSA NSPs)-ninthedanib	NHS-ester of FA	BSA	saturated NaHCO_3_ aqueous solution; incubation for 24 h	ninthedanib	-	Modification rate for BSA in NSPs with folate is 28.1%	-	Fluorescence spectroscopy using BSA with incorporated fluorescent α-amino acids BSA and fluorescent aminoacetone	+	-	-	[24]
folate-(BSA-containing NSPs)-doxorubicin	NHS-ester of FA	BSA-containing magnetic iron oxide NSPs with doxorubicin	carbonate/bicarbonate buffer (pH 10.0); incubation for 2 h	-	Presence of folate was identified by characteristic peak of FA in BSA-containing magnetic iron oxide NSPs with doxorubicin	-	-	-	+	-	-	[31]
folate-(HSA NSPs)-Curcumin	NHS-ester of FA	HSA NSPs with Curcumin	carbonate/bicarbonate buffer (0.2 M, pH 10); incubation for 1 h	-	-	-	-	-	-	-	+	[17]
folate-(HSA NSPs)-(fluorescein isothiocyanate)	NHS-ester of FA	HSA NSPs wit fluorescein isothiocyanate	carbonate/bicarbonate buffer (0.13 M, pH 7); incubation for 12 h	-	The folate concentration was determined by calculating the area of the absorbance peak and interpolation using a calibration curve of FA in the presence of HSA NSPs as background	0.38:1 for 10/1 molar ratio between FA and HSA; 6.42:1 for 50/1 molar ratio between FA and HSA	-	-	-	-	-	[45]
folate-(BSA-containing NSPs)-fluorescein isothiocyanate	NHS-ester of FA	BSA-containing superparamagnetic iron oxide NSPs	MES buffer (pH 5.5); incubation for 2 h; darkness	fluorescein isothiocyanate	-	-	-	-	+	-	-	[38]
folate-(HSA NSPs)-doxorubicin	FA	HSA NSPs	water; incubation for 1 min	doxorubicin	Presence of folate was identified by characteristic peak of FA in folate-(HSA NSPs)-doxorubicin	-	+	-	-	-	-	[107]
folate-(HSA-containing NSPs)-methylene blue	NHS-ester of FA	HSA or HSA conjugated with Cyanine 5 NHS-ester containing magnetic iron oxide nanoparticles	25 mM phosphate buffer (pH 7.4) with 25% DMSO; overnight incubation	methylene blue	Presence of folate was identified by characteristic peak of FA in folate-HSA	-	-	Fluorescence spectroscopy	+	+	-	[132]

List of abbreviations: DMSO—dimethyl sulfoxide; EDC—(N-(3-dimethylaminopropyl)-N-ethylcarbodiimide; FR−—FR-negative cells; FR+– FR-positive cells; IR-780—2-[2-[2-Chloro-3-[(1,3-dihydro-3,3-dimethyl-1-propyl-2H-indol-2-ylidene)ethylidene]-1-cyclohexen-1-yl]ethenyl]-3,3-dimethyl-1-propylindolium; SPIONs—superparamagnetic iron oxide nanoparticles; UV-Vis—UV-Vis spectroscopy. “-“ means that there is absence of data in the study to fill the cell of the table; “+” means that there is data in the study obtained using the method to which the column is devoted.

**Table 2:** Binding constants of albumins with folic acid assessed by fluorescence spectroscopy and calculated using Stern-Volmer equation variations.

Albumin	Binding Parameters				Medium and Temperature (°C)	References
	K_SV_, 10^4^ (L*mol^−1^)	K_Q_, 10^12^ (L*mol^−1^*s^−1^)	K_a_ or K_b_, 10^4^ (L*mol^−1^)	n		
BSA	7.58	8.94	20.40	1.04	Tris-HCl buffer (pH 7.4) with NaCl; 20 °C	[114]
BSA	9.11 ± 0.02	13.20	8.72 ± 0.19	–	HEPES buffer (pH 7.25); 25 °C	[111]
BSA	1.55	100.00	–	–	phosphate buffer (pH 7.0)	[113]
BSA	10.00 ± 0.30	–	–	–	Tris-HCl buffer (pH = 7.4); 24 °C	[109,110]
HSA	8.10 ± 0.80	–	–	–		
HSA	0.282 ± 0.03	0.408 ± 0.004	0.62 ± 0.09	1.20	glycine buffer (pH 7.4); 25 °C	[186]
HSA	8.97	13.50	9.78	1.22	HEPES buffer (pH 7.25); 25 °C	[112]
HSA	7.77 ± 0.21	–	–	1.09 ± 0.1	phosphate buffer (pH 7.4); 26.85 °C	[108]
HSA	2.58	2.58	–	–	PBS (pH 7.4); 24.85 °C	[180]

List of abbreviations: K_Q_ is the quenching rate constant of a bimolecule; K_sv_ is the dynamic quenching constant; K_a_ and K_b_ are binding constants; n is the number of binding sites; “–“ means that there is absence of data in the study to fill the cell of the table

## Data Availability

No new data were created or analyzed in this study.

## Abbreviations

The following abbreviations are used in this manuscript:

BSA	bovine serum albumin
CKD	of chronic kidney disease
DCC	N,N’-dicyclohexylcarbodiimide
Dir	dimethylindole red
DMSO	dimethyl sulfoxide
DSC	differential scanning calorimetry
EDAC (or EDCI)	1-ethyl-3-(3-dimethylaminopropyl) carbodiimide
EDC	(N-(3-dimethylaminopropyl)-N-ethylcarbodiimide
EPR	effect of enhanced permeability and retention
FA	Folic acid
FR	folate (folic acid) receptors
FR-	FR-negative cells
FR+	FR-positive cells
FS	fluorescence spectroscopy
FTIR	Fourier transform infrared spectroscopy
GP60	glycoprotein 60 receptor
HPLC	high-performance liquid chromatography
HAS	human serum albumin
IR-780	2-[2-[2-Chloro-3-[(1,3-dihydro-3,3-dimethyl-1-propyl-2H-indol-2-ylidene)ethylidene]-1-cyclohexen-1-yl]ethenyl]-3,3-dimethyl-1-propylindolium
MRI	magnetic resonance imaging
NHS	N-hydroxysuccinimide
NMR	Nuclear magnetic resonance
NSPs	nano-and submicron particles
PEG	polyethylene glycol
PDT	photodynamic therapy
PTT	photothermal therapy
SEC	size exclusion chromatography
SPARC	secreted protein acidic and rich in cysteine
SPIONs	superparamagnetic iron oxide nanoparticles
SPR	surface plasmon resonance
UV-MALDI-TOF MS	ultraviolet matrix-assisted laser desorption/ionization time-of-flight mass spectrometry
UV-Vis	UV-Vis spectroscopy
VBLS	vinblastine sulfate

## References

[1] Moens A.L., Claeys M.J., Wuyts F.L., Goovaerts I., Van Hertbruggen E., Wendelen L.C., Van Hoof V.O., Vrints C.J. (2007). Effect of Folic Acid on Endothelial Function Following Acute Myocardial Infarction. Am. J. Cardiol..

[2] Xu X., Qin X., Li Y., Sun D., Wang J., Liang M., Wang B., Huo Y., Hou F.F., Cao K. (2016). Efficacy of Folic Acid Therapy on the Progression of Chronic Kidney Disease: The Renal Substudy of the China Stroke Primary Prevention Trial. JAMA Intern. Med..

[3] Boshnjaku V., Shim K.W., Tsurubuchi T., Ichi S., Szany E.V., Xi G., Mania-Farnell B., McLone D.G., Tomita T., Mayanil C.S. (2012). Nuclear Localization of Folate Receptor Alpha: A New Role as a Transcription Factor. Sci. Rep..

[4] Argyridis S. (2019). Folic Acid in Pregnancy. Obstet. Gynaecol. Reprod. Med..

[5] Jurczyk M., Jelonek K., Musiał-kulik M., Beberok A., Wrześniok D., Kasperczyk J. (2021). Single- versus Dual-targeted Nanoparticles with Folic Acid and Biotin for Anticancer Drug Delivery. Pharmaceutics.

[6] Ebrahimnejad P., Sodagar Taleghani A., Asare-Addo K., Nokhodchi A. (2022). An Updated Review of Folate-Functionalized Nanocarriers: A Promising Ligand in Cancer. Drug Discov. Today.

[7] Bahrami B., Mohammadnia-Afrouzi M., Bakhshaei P., Yazdani Y., Ghalamfarsa G., Yousefi M., Sadreddini S., Jadidi-Niaragh F., Hojjat-Farsangi M. (2015). Folate-Conjugated Nanoparticles as a Potent Therapeutic Approach in Targeted Cancer Therapy. Tumor Biol..

[8] Narmani A., Rezvani M., Farhood B., Darkhor P., Mohammadnejad J., Amini B., Refahi S., Abdi Goushbolagh N. (2019). Folic Acid Functionalized Nanoparticles as Pharmaceutical Carriers in Drug Delivery Systems. Drug Dev. Res..

[9] Gorobets M.G., Toroptseva A.V., Abdullina M.I., Pokrovsky V.S., Khachatryan D.S., Bychkova A.V. (2025). Folic Acid Conjugated with Serum Albumin for Nano- and Submicron Delivery Systems for Applications in Therapy and Diagnostics. Explor. Drug Sci..

[10] Yan S., Na J., Liu X., Wu P. (2024). Different Targeting Ligands-Mediated Drug Delivery Systems for Tumor Therapy. Pharmaceutics.

[11] Farber S., Cutler E.C., Hawkins J.W., Harrison J.H., Peirce E.C., Lenz G.G. (1947). The Action of Pteroylglutamic Conjugates on Man. Science.

[12] Chen C., Hu H., Qiao M., Zhao X., Wang Y., Chen K., Guo X., Chen D. (2015). Tumor-Targeting and PH-Sensitive Lipoprotein-Mimic Nanocarrier for Targeted Intracellular Delivery of Paclitaxel. Int. J. Pharm..

[13] Ma N., Liu J., He W., Li Z., Luan Y., Song Y., Garg S. (2017). Folic Acid-Grafted Bovine Serum Albumin Decorated Graphene Oxide: An Efficient Drug Carrier for Targeted Cancer Therapy. J. Colloid Interface Sci..

[14] Shen Z., Li Y., Kohama K., Oneill B., Bi J. (2011). Improved Drug Targeting of Cancer Cells by Utilizing Actively Targetable Folic Acid-Conjugated Albumin Nanospheres. Pharmacol. Res..

[15] Solanki R., Srivastav A.K., Patel S., Singh S.K., Jodha B., Kumar U., Patel S. (2024). Folate Conjugated Albumin as a Targeted Nanocarrier for the Delivery of Fisetin: In Silico and In Vitro Biological Studies. RSC Adv..

[16] Zhao D., Zhao X., Zu Y., Li J., Zhang Y., Jiang R., Zhang Z. (2010). Preparation, Characterization, and In Vitro Targeted Delivery of Folate-Decorated Paclitaxel-Loaded Bovine Serum Albumin Nanoparticles. Int. J. Nanomed..

[17] Song Z., Lu Y., Zhang X., Wang H., Han J., Dong C. (2016). Novel Curcumin-Loaded Human Serum Albumin Nanoparticles Surface Functionalized with Folate: Characterization and in Vitro/Vivo Evaluation. Drug Des. Devel. Ther..

[18] Bardania H., Jafari F., Baneshi M., Mahmoudi R., Ardakani M.T., Safari F., Barmak M.J. (2023). Folic Acid-Functionalized Albumin/Graphene Oxide Nanocomposite to Simultaneously Deliver Curcumin and 5-Fluorouracil into Human Colorectal Cancer Cells: An in Vitro Study. BioMed Res. Int..

[19] Nosrati H., Abbasi R., Charmi J., Rakhshbahar A., Aliakbarzadeh F., Danafar H., Davaran S. (2018). Folic Acid Conjugated Bovine Serum Albumin: An Efficient Smart and Tumor Targeted Biomacromolecule for Inhibition Folate Receptor Positive Cancer Cells. Int. J. Biol. Macromol..

[20] Nateghian N., Goodarzi N., Amini M., Atyabi F., Khorramizadeh M.R., Dinarvand R. (2016). Biotin/Folate-Decorated Human Serum Albumin Nanoparticles of Docetaxel: Comparison of Chemically Conjugated Nanostructures and Physically Loaded Nanoparticles for Targeting of Breast Cancer. Chem. Biol. Drug Des..

[21] Li Y., Wang T., Liu Y., Xu Y., Sun Z., Yang G. (2019). NIR-Laser-Triggered Drug Release from Folate Decorated Albumin Nanoparticles for Synergistic Chemo-Photothermal Tumor Therapy. J. Drug Deliv. Sci. Technol..

[22] Hiremath C.G., Kariduraganavar M.Y., Hiremath M.B. (2018). Synergistic Delivery of 5-Fluorouracil and Curcumin Using Human Serum Albumin-Coated Iron Oxide Nanoparticles by Folic Acid Targeting. Prog. Biomater..

[23] Meng F., Liu F., Lan M., Zou T., Li L., Cai T., Cai Y. (2021). Preparation and Evaluation of Folate-Modified Albumin Baicalin-Loaded Nanoparticles for the Targeted Treatment of Breast Cancer. J. Drug Deliv. Sci. Technol..

[24] Zha Q., Zhang L., Guo Y., Bao R., Shi F., Shi Y. (2022). Preparation and Study of Folate Modified Albumin Targeting Microspheres. J. Oncol..

[25] Dubey R.D., Alam N., Saneja A., Khare V., Kumar A., Vaidh S., Mahajan G., Sharma P.R., Singh S.K., Mondhe D.M. (2015). Development and Evaluation of Folate Functionalized Albumin Nanoparticles for Targeted Delivery of Gemcitabine. Int. J. Pharm..

[26] Du C., Deng D., Shan L., Wan S., Cao J., Tian J., Achilefu S., Gu Y. (2013). A PH-Sensitive Doxorubicin Prodrug Based on Folate-Conjugated BSA for Tumor-Targeted Drug Delivery. Biomaterials.

[27] Butzbach K., Rasse-Suriani F.A.O., Gonzalez M.M., Cabrerizo F.M., Epe B. (2016). Albumin-Folate Conjugates for Drug-Targeting in Photodynamic Therapy. Photochem. Photobiol..

[28] Dehvari K., Li J.D., Chang J.Y. (2019). Bovine Serum Albumin-Templated Synthesis of Manganese-Doped Copper Selenide Nanoparticles for Boosting Targeted Delivery and Synergistic Photothermal and Photodynamic Therapy. ACS Appl. Bio Mater..

[29] Girma W.M., Dehvari K., Ling Y.C., Chang J.Y. (2019). Albumin-Functionalized CuFeS 2/Photosensitizer Nanohybrid for Single-Laser-Induced Folate Receptor-Targeted Photothermal and Photodynamic Therapy. Mater. Sci. Eng. C.

[30] Li J., Cai R., Kawazoe N., Chen G. (2015). Facile Preparation of Albumin-Stabilized Gold Nanostars for the Targeted Photothermal Ablation of Cancer Cells. J. Mater. Chem. B.

[31] Yang R., An Y.L., Miao F.Q., Li M.F., Liu P.D., Tang Q.S. (2014). Preparation of Folic Acid-Conjugated, Doxorubicin-Loaded, Magnetic Bovine Serum Albumin Nanospheres and Their Antitumor Effects in Vitro and in Vivo. Int. J. Nanomed..

[32] Lee H., Kim S., Oh C., Khan I., Shukla S., Bajpai V.K., Han Y.K., Huh Y.S. (2020). Folic Acid-Modified Bovine Serum Albumin Nanoparticles with Doxorubicin and Chlorin E6 for Effective Combinational Chemo-Photodynamic Therapy. Mater. Sci. Eng. C.

[33] Encinas-Basurto D., Ibarra J., Juarez J., Pardo A., Barbosa S., Taboada P., Valdez M.A. (2018). Hybrid Folic Acid-Conjugated Gold Nanorods-Loaded Human Serum Albumin Nanoparticles for Simultaneous Photothermal and Chemotherapeutic Therapy. Mater. Sci. Eng. C.

[34] Zhang Y., Liang Y. (2024). Fabrication of Folic Acid-Modified Bovine Serum Albumin Cloaked Dual-Drug Loaded Hollow Mesoporous Silica Nanoparticles for PH-Responsive and Targeted Delivery of Gastric Cancer Therapy. Heliyon.

[35] Zha S., Liu H., Li H., Li H., Wong K.L., All A.H. (2024). Functionalized Nanomaterials Capable of Crossing the Blood-Brain Barrier. ACS Nano.

[36] Xu L., Jiang G., Chen H., Zan Y., Hong S., Zhang T., Zhang Y., Pei R. (2019). Folic Acid-Modified Fluorescent Dye-Protein Nanoparticles for the Targeted Tumor Cell Imaging. Talanta.

[37] Li H., Yan K., Shang Y., Shrestha L., Liao R., Liu F., Li P., Xu H., Xu Z., Chu P.K. (2015). Folate-Bovine Serum Albumin Functionalized Polymeric Micelles Loaded with Superparamagnetic Iron Oxide Nanoparticles for Tumor Targeting and Magnetic Resonance Imaging. Acta Biomater..

[38] Wang X., Tu M., Tian B., Yi Y., Wei Z.Z., Wei F. (2016). Synthesis of Tumor-Targeted Folate Conjugated Fluorescent Magnetic Albumin Nanoparticles for Enhanced Intracellular Dual-Modal Imaging into Human Brain Tumor Cells. Anal. Biochem..

[39] Li F., Yang G., Aguilar Z.P., Xiong Y., Xu H. (2018). Affordable and Simple Method for Separating and Detecting Ovarian Cancer Circulating Tumor Cells Using BSA Coated Magnetic Nanoprobes Modified with Folic Acid. Sens. Actuators B Chem..

[40] Dey C., Ghosh A., Ahir M., Ghosh A., Mandal Goswami M. (2018). Improvement of Anticancer Drug Release by Cobalt Ferrite Magnetic Nanoparticles through Combined PH and Temperature Responsive Technique. ChemPhysChem.

[41] Ayala-López W., Xia W., Varghese B., Low P.S. (2010). Imaging of Atherosclerosis in Apoliprotein e Knockout Mice: Targeting of a Folate-Conjugated Radiopharmaceutical to Activated Macrophages. J. Nucl. Med..

[42] Dave V., Sharma R., Gupta C., Sur S. (2020). Folic Acid Modified Gold Nanoparticle for Targeted Delivery of Sorafenib Tosylate towards the Treatment of Diabetic Retinopathy. Colloids Surf. B Biointerfaces.

[43] Hartmann L.C., Keeney G.L., Lingle W.L., Christianson T.J.H., Varghese B., Hillman D., Oberg A.L., Low P.S. (2007). Folate Receptor Overexpression Is Associated with Poor Outcome in Breast Cancer. Int. J. Cancer.

[44] Nogueira E., Gomes A.C., Preto A., Cavaco-Paulo A. (2016). Folate-Targeted Nanoparticles for Rheumatoid Arthritis Therapy. Nanomed. Nanotechnol. Biol. Med..

[45] Rollett A., Reiter T., Nogueira P., Cardinale M., Loureiro A., Gomes A., Cavaco-Paulo A., Moreira A., Carmo A.M., Guebitz G.M. (2012). Folic Acid-Functionalized Human Serum Albumin Nanocapsules for Targeted Drug Delivery to Chronically Activated Macrophages. Int. J. Pharm..

[46] Bilthariya U., Jain N., Rajoriya V., Jain A.K. (2015). Folate-Conjugated Albumin Nanoparticles for Rheumatoid Arthritis-Targeted Delivery of Etoricoxib. Drug Dev. Ind. Pharm..

[47] Jin Y., Zhang Q., Qin X., Liu Z., Li Z., Zhong X., Xia L., He J., Fang B. (2022). Carbon Dots Derived from Folic Acid Attenuates Osteoarthritis by Protecting Chondrocytes through NF-ΚB/MAPK Pathway and Reprogramming Macrophages. J. Nanobiotechnology.

[48] Kunde S.S., Wairkar S. (2022). Targeted Delivery of Albumin Nanoparticles for Breast Cancer: A Review. Colloids Surf. B Biointerfaces.

[49] Peters T. (1995). All About Albumin.

[50] Quinlan G.J., Martin G.S., Evans T.W. (2005). Albumin: Biochemical Properties and Therapeutic Potential. Hepatology.

[51] Zu Y., Zhang Y., Zhao X., Zhang Q., Liu Y., Jiang R. (2009). Optimization of the Preparation Process of Vinblastine Sulfate (VBLS)-Loaded Folate-Conjugated Bovine Serum Albumin (BSA) Nanoparticles for Tumor-Targeted Drug Delivery Using Response Surface Methodology (RSM). Int. J. Nanomed..

[52] Al-Rahim A.M., AlChalabi R., Al-Saffar A.Z., Sulaiman G.M., Albukhaty S., Belali T., Ahmed E.M., Khalil K.A.A. (2023). Folate-Methotrexate Loaded Bovine Serum Albumin Nanoparticles Preparation: An in Vitro Drug Targeting Cytokines Overwhelming Expressed Immune Cells from Rheumatoid Arthritis Patients. Anim. Biotechnol..

[53] Zhu Y., Li C., Yu J., Yu L., Shao W., Shang S. (2022). Interaction of Remimazolam Benzenesulfonate and Human Serum Albumin: A Simulated Physiological Study. Luminescence.

[54] Cheng L., Niu M.M., Yan T., Ma Z., Huang K., Yang L., Zhong X., Li C. (2021). Bioresponsive Micro-to-Nano Albumin-Based Systems for Targeted Drug Delivery against Complex Fungal Infections. Acta Pharm. Sin. B.

[55] Turcsányi Á., Ungor D., Csapó E. (2020). Fluorescent Labeling of Hyaluronic Acid-Chitosan Nanocarriers by Protein-Stabilized Gold Nanoclusters. Crystals.

[56] Phan V.H.G., Le T.M.D., Janarthanan G., Ngo P.K.T., Lee D.S., Thambi T. (2021). Development of Bioresorbable Smart Injectable Hydrogels Based on Thermo-Responsive Copolymer Integrated Bovine Serum Albumin Bioconjugates for Accelerated Healing of Excisional Wounds. J. Ind. Eng. Chem..

[57] Barmpa A., Hatzidimitriou A.G., Psomas G. (2021). Copper(II) Complexes with Meclofenamate Ligands: Structure, Interaction with DNA and Albumins, Antioxidant and Anticholinergic Activity. J. Inorg. Biochem..

[58] Barmpa A., Geromichalos G.D., Hatzidimitriou A.G., Psomas G. (2021). Nickel(II)–Meclofenamate Complexes: Structure, in Vitro and in Silico DNA– and Albumin–Binding Studies, Antioxidant and Anticholinergic Activity. J. Inorg. Biochem..

[59] Nathasia, Tansil T.S. (2020). Albumin, Important Therapy & When to Use It in Ten Patients (Adult & Child): Case Report. J. Dermatol. Res. Ther..

[60] Caraceni P., Tufoni M., Bonavita M.E. (2013). Clinical Use of Albumin. Blood Transfus..

[61] Ulbrich K., Michaelis M., Rothweiler F., Knobloch T., Sithisarn P., Cinatl J., Kreuter J. (2011). Interaction of Folate-Conjugated Human Serum Albumin (HSA) Nanoparticles with Tumour Cells. Int. J. Pharm..

[62] Tenchov R., Hughes K.J., Ganesan M., Iyer K.A., Ralhan K., Lotti Diaz L.M., Bird R.E., Ivanov J.M., Zhou Q.A. (2025). Transforming Medicine: Cutting-Edge Applications of Nanoscale Materials in Drug Delivery. ACS Nano.

[63] Stanger O. (2005). Physiology of Folic Acid in Health and Disease. Curr. Drug Metab..

[64] Liang L. (2020). Folates: Stability and Interaction with Biological Molecules. J. Agric. Food Res..

[65] Soliman H.A., Olesen H. (1976). Folic Acid Binding by Human Plasma Albumin. Scand. J. Clin. Lab. Investig..

[66] Markert S., Lassmann S., Gabriel B., Klar M., Werner M., Gitsch G., Kratz F., Hasenburg A. (2008). Alpha-Folate Receptor Expression in Epithelial Ovarian Carcinoma and Non-Neoplastic Ovarian Tissue. Anticancer Res..

[67] Young O., Ngo N., Lin L., Stanbery L., Creeden J.F., Hamouda D., Nemunaitis J. (2023). Folate Receptor as a Biomarker and Therapeutic Target in Solid Tumors. Curr. Probl. Cancer.

[68] Trindade A.F., Frade R.F.M., MaçÔas E.M.S., Graça C., Rodrigues C.A.B., Martinho J.M.G., Afonso C.A.M. (2014). “click and Go”: Simple and Fast Folic Acid Conjugation. Org. Biomol. Chem..

[69] Chen C., Ke J., Edward Zhou X., Yi W., Brunzelle J.S., Li J., Yong E.L., Xu H.E., Melcher K. (2013). Structural Basis for Molecular Recognition of Folic Acid by Folate Receptors. Nature.

[70] Wibowo A.S., Singh M., Reeder K.M., Carter J.J., Kovach A.R., Meng W., Ratnam M., Zhang F., Dann C.E. (2013). Structures of Human Folate Receptors Reveal Biological Trafficking States and Diversity in Folate and Antifolate Recognition. Proc. Natl. Acad. Sci. USA.

[71] Figliola C., Marchal E., Groves B.R., Thompson A. (2019). A Step-Wise Synthetic Approach Is Necessary to Access γ-Conjugates of Folate: Folate-Conjugated Prodigiosenes. RSC Adv..

[72] Wang S., Low P.S. (1998). Folate-Mediated Targeting of Antineoplastic Drugs, Imaging Agents, and Nucleic Acids to Cancer Cells. J. Control. Release.

[73] Carron P.M., Crowley A., O’Shea D., McCann M., Howe O., Hunt M., Devereux M. (2018). Targeting the Folate Receptor: Improving Efficacy in Inorganic Medicinal Chemistry. Curr. Med. Chem..

[74] Boss S.D., Betzel T., Müller C., Fischer C.R., Haller S., Reber J., Groehn V., Schibli R., Ametamey S.M. (2016). Comparative Studies of Three Pairs of α- And γ-Conjugated Folic Acid Derivatives Labeled with Fluorine-18. Bioconjugate Chem..

[75] Scaranti M., Cojocaru E., Banerjee S., Banerji U. (2020). Exploiting the Folate Receptor α in Oncology. Nat. Rev. Clin. Oncol..

[76] Moazzen S., Dastgiri S., Dolatkhah R., Abdolahi H.M., Alizadeh B.Z., de Bock G.H. (2020). Folic Acid Supplement Intake and Risk of Colorectal Cancer in Women; a Case Control Study. Ann. Glob. Health.

[77] Qin T., Du M., Du H., Shu Y., Wang M., Zhu L. (2015). Folic Acid Supplements and Colorectal Cancer Risk: Meta-Analysis of Randomized Controlled Trials. Sci. Rep..

[78] Burr N.E., Hull M.A., Subramanian V. (2017). Folic Acid Supplementation May Reduce Colorectal Cancer Risk in Patients With Inflammatory Bowel Disease. J. Clin. Gastroenterol..

[79] D’Angelica M., Ammori J., Gonen M., Klimstra D.S., Low P.S., Murphy L., Weiser M.R., Paty P.B., Fong Y., Dematteo R.P. (2011). Folate Receptor-α Expression in Resectable Hepatic Colorectal Cancer Metastases: Patterns and Significance. Mod. Pathol..

[80] Paliwal S., Jana P., Singh S., Madhyastha H., Webster T.J., Dev A. (2022). Folic Acid Conjugated Capecitabine Capped Green Synthesized Fluorescent Carbon Dots as a Targeted Nano-Delivery System for Colorectal Cancer. Mater. Today Commun..

[81] Omote S., Takata K., Tanaka T., Miyata-Takata T., Ayada Y., Noujima-Harada M., Omote R., Tabata T., Sato Y., Toyokawa T. (2018). Overexpression of Folate Receptor Alpha Is an Independent Prognostic Factor for Outcomes of Pancreatic Cancer Patients. Med. Mol. Morphol..

[82] Liu C., Ding L., Bai L., Chen X., Kang H., Hou L., Wang J. (2017). Folate Receptor Alpha Is Associated with Cervical Carcinogenesis and Regulates Cervical Cancer Cells Growth by Activating ERK1/2/c-Fos/c-Jun. Biochem. Biophys. Res. Commun..

[83] Allard J.E., Risinger J.I., Morrison C., Young G., Rose G.S., Fowler J., Berchuck A., Maxwell G.L. (2007). Overexpression of Folate Binding Protein Is Associated with Shortened Progression-Free Survival in Uterine Adenocarcinomas. Gynecol. Oncol..

[84] Senol S., Ceyran A.B., Aydin A., Zemheri E., Ozkanli S., Kösemetin D., Sehitoglu I., Akalin I. (2015). Folate Receptor α Expression and Significance in Endometrioid Endometrium Carcinoma and Endometrial Hyperplasia. Int. J. Clin. Exp. Pathol..

[85] O’shannessy D.J., Somers E.B., Smale R., Fu Y.S. (2013). Expression of Folate Receptor-α (FRA) in Gynecologic Malignancies and Its Relationship to the Tumor Type. Int. J. Gynecol. Pathol..

[86] Akal Z.U., Alpsoy L., Baykal A. (2016). Superparamagnetic Iron Oxide Conjugated with Folic Acid and Carboxylated Quercetin for Chemotherapy Applications. Ceram. Int..

[87] Monteiro C.A.P., Oliveira A.D.P.R., Silva R.C., Lima R.R.M., Souto F.O., Baratti M.O., Carvalho H.F., Santos B.S., Cabral Filho P.E., Fontes A. (2020). Evaluating Internalization and Recycling of Folate Receptors in Breast Cancer Cells Using Quantum Dots. J. Photochem. Photobiol. B Biol..

[88] Zhang Z., Wang J., Tacha D.E., Li P., Bremer R.E., Chen H., Wei B., Xiao X., Da J., Skinner K. (2014). Folate Receptor α Associated with Triple-Negative Breast Cancer and Poor Prognosis. Arch. Pathol. Lab. Med..

[89] Nutt J.E., Razak A.R.A., O’Toole K., Black F., Quinn A.E., Calvert A.H., Plummer E.R., Lunec J. (2010). The Role of Folate Receptor Alpha (FRα) in the Response of Malignant Pleural Mesothelioma to Pemetrexed-Containing Chemotherapy. Br. J. Cancer.

[90] Saba N.F., Wang X., Müller S., Tighiouart M., Cho K., Nie S., Chen Z., Shin D.M. (2009). Examining Expression of Folate Receptor in Squamous Cell Carcinoma of the Head and Neck as a Target for a Novel Nanotherapeutic Drug. Head Neck.

[91] Zhang H., Li J., Hu Y., Shen M., Shi X., Zhang G. (2016). Folic Acid-Targeted Iron Oxide Nanoparticles as Contrast Agents for Magnetic Resonance Imaging of Human Ovarian Cancer. J. Ovarian Res..

[92] Vakhrusheva T.V., Gusev A.A., Gusev S.A., Vlasova I.I. (2013). Albumin Reduces Thrombogenic Potential of Single-Walled Carbon Nanotubes. Toxicol. Lett..

[93] Chubarov A.S. (2022). Serum Albumin for Magnetic Nanoparticles Coating. Magnetochemistry.

[94] Jia L., Guo L., Zhu J., Ma Y. (2014). Stability and Cytocompatibility of Silk Fibroin-Capped Gold Nanoparticles. Mater. Sci. Eng. C.

[95] Chou H.C., Chiu S.J., Hu T.M. (2015). LbL Assembly of Albumin on Nitric Oxide-Releasing Silica Nanoparticles Using Suramin, a Polyanion Drug, as an Interlayer Linker. Biomacromolecules.

[96] Quan Q., Xie J., Gao H., Yang M., Zhang F., Liu G., Lin X., Wang A., Eden H.S., Lee S. (2011). HSA Coated Iron Oxide Nanoparticles as Drug Delivery Vehicles for Cancer Therapy. Mol. Pharm..

[97] Song J., Fransen P.P.K.H., Bakker M.H., Wijnands S.P.W., Huang J., Guo S., Dankers P.Y.W. (2024). The Effect of Charge and Albumin on Cellular Uptake of Supramolecular Polymer Nanostructures. J. Mater. Chem. B.

[98] Lindner J.L., Loibl S., Denkert C., Ataseven B., Fasching P.A., Pfitzner B.M., Gerber B., Gade S., Darb-Esfahani S., Sinn B.V. (2015). Expression of Secreted Protein Acidic and Rich in Cysteine (SPARC) in Breast Cancer and Response to Neoadjuvant Chemotherapy. Ann. Oncol..

[99] Cho H., Jeon S.I., Ahn C.H., Shim M.K., Kim K. (2022). Emerging Albumin-Binding Anticancer Drugs for Tumor-Targeted Drug Delivery: Current Understandings and Clinical Translation. Pharmaceutics.

[100] An J.M., Moon H., Verwilst P., Shin J., Kim B.M., Park C., Kim J.S., Yeo S.G., Kim H.Y., Kim D. (2021). Human Glioblastoma Visualization: Triple Receptor-Targeting Fluorescent Complex of Dye, SIWV Tetra-Peptide, and Serum Albumin Protein. ACS Sens..

[101] Patel K., Jain P., Kumar P., Kumar A., Solanki R. (2022). Human Serum Albumin-Based Propulsive Piperlongumine-Loaded Nanoparticles: Formulation Development, Characterization and Anti-Cancer Study. Colloids Surf. A Physicochem. Eng. Asp..

[102] Wu J. (2021). The Enhanced Permeability and Retention (EPR) Effect: The Significance of the Concept and Methods to Enhance Its Application. J. Pers. Med..

[103] Deng M., Li M. (2021). Size-Adjustable Nano-Drug Delivery Systems for Enhanced Tumor Retention and Penetration. Pharm. Front..

[104] Toroptseva A.V., Markova A.A., Nguyen M.T., Abdullina M.I., Motyakin M.V., Mayorova O.A., Bychkova A.V. (2025). Nanosystems Consisting of Iron Oxide and Serum Albumin As a Platform for Drug Delivery into Cells: Analysis of the Cy5-HSA@IONPs Nanosystem Components before and after Accumulation by Tumor Cells. Biochem. Suppl. Ser. A Membr. Cell Biol..

[105] Kaur S., Singh M., Brkljaca R., Anderson S.R., Korte J., Svoboda P., Mašková-Černá S., Urban S., Shukla R., Ramanathan R. (2024). Artificial Magnetosomes: Molecularly Restructured SPIONs with Enhanced Potential for Magnetic Imaging. Mater. Today Chem..

[106] Xu X., Zhang Y., Meng C., Zheng W., Wang L., Zhao C., Luo F. (2024). Nanozymes in Cancer Immunotherapy: Metabolic Disruption and Therapeutic Synergy. J. Mater. Chem. B.

[107] Khalil K.A.A., Al-Musawi S., Albukhaty S., Sulaiman G.M., Al-Karagoly H., Ahmed E.M., Solaiman M.T. (2021). Formulation of Folate-Conjugated, Doxorubicin-Loaded Human Serum Albumin Nanoparticles for Promotion of Gene Expression Associated with Apoptosis in Renal Cell Carcinoma. Res. Sq..

[108] Śliwińska-Hill U., Wiglusz K. (2019). Multispectroscopic Studies of the Interaction of Folic Acid with Glycated Human Serum Albumin. J. Biomol. Struct. Dyn..

[109] Bourassa P., Hasni I., Tajmir-Riahi H.A. (2011). Folic Acid Complexes with Human and Bovine Serum Albumins. Food Chem..

[110] Bourassa P., Chanphai P., Tajmir-Riahi H.A. (2017). Folic Acid Delivery by Serum Proteins: Loading Efficacy and Protein Morphology. J. Biomol. Struct. Dyn..

[111] Chilom C.G., David M., Florescu M. (2020). Monitoring Biomolecular Interaction Between Folic Acid and Bovine Serum Albumin. Spectrochim. Acta-Part A Mol. Biomol. Spectrosc..

[112] Chilom C.G., Bacalum M., Stanescu M.M., Florescu M. (2018). Insight into the Interaction of Human Serum Albumin with Folic Acid: A Biophysical Study. Spectrochim. Acta-Part A Mol. Biomol. Spectrosc..

[113] Jha N.S., Kishore N. (2011). Thermodynamic Studies on the Interaction of Folic Acid with Bovine Serum Albumin. J. Chem. Thermodyn..

[114] Zhang A., Jia L. (2006). Spectroscopic Study of the Interaction Between Folic Acid and Bovine Serum Albumin. Spectrosc. Lett..

[115] Jo S.M., Noh S.H., Jin Z., Lim Y., Cheon J., Kim H.S. (2014). Simple and Efficient Capture of EGFR-Expressing Tumor Cells Using Magnetic Nanoparticles. Sens. Actuators B Chem..

[116] Movileanu C., Anghelache M., Turtoi M., Voicu G., Neacsu I.A., Ficai D., Trusca R., Oprea O., Ficai A., Andronescu E. (2022). Folic Acid-Decorated PEGylated Magnetite Nanoparticles as Efficient Drug Carriers to Tumor Cells Overexpressing Folic Acid Receptor. Int. J. Pharm..

[117] Coelho C.D.F., Paiva V.S., Almeida Z.L., Jesus J.A., Marteleira M., Ramos C.V., Cruz P.F., Costa T., Moura C.S., Trindade D. (2025). Serum-PEG and BSA-PEG Hydrogels as Advanced Platforms for Evaluating Plasma Protein Binding. Mater. Today Chem..

[118] Al-Salih M.Y.A., Pouresmaeil V., Davoodi-Dehaghani F., Haghighi H.N., Tabrizi M.H. (2023). Study the Anticancer Properties of Thymol-Loaded PEGylated Bovine Serum Albumin Nanoparticles Conjugated with Folic Acid. Chem. Biodivers..

[119] Xie J., Xu C., Kohler N., Hou Y., Sun S. (2007). Controlled PEGylation of Monodisperse Fe3O4 Nanoparticles for Reduced Non-Specific Uptake by Macrophage Cells. Adv. Mater..

[120] Otsuka H., Nagasaki Y., Kataoka K. (2003). PEGylated Nanoparticles for Biological and Pharmaceutical Applications. Adv. Drug Deliv. Rev..

[121] Zou Z., He X., He D., Wang K., Qing Z., Yang X., Wen L., Xiong J., Li L., Cai L. (2015). Programmed Packaging of Mesoporous Silica Nanocarriers for Matrix Metalloprotease 2-Triggered Tumor Targeting and Release. Biomaterials.

[122] Firouzabadi K., Karimi E., Tabrizi M.H. (2023). Fabrication of Bovine Serum Albumin-Polyethylene Glycol Nanoparticle Conjugated-Folic Acid Loaded-Naringenin as an Efficient Carrier Biomacromolecule for Suppression of Cancer Cells. Biotechnol. Appl. Biochem..

[123] Siwowska K., Haller S., Bortoli F., Benešová M., Groehn V., Bernhardt P., Schibli R., Müller C. (2017). Preclinical Comparison of Albumin-Binding Radiofolates: Impact of Linker Entities on the in Vitro and in Vivo Properties. Mol. Pharm..

[124] Elzoghby A.O., Hemasa A.L., Freag M.S. (2016). Hybrid Protein-Inorganic Nanoparticles: From Tumor-Targeted Drug Delivery to Cancer Imaging. J. Control. Release.

[125] Chu Y., Chai S., Pan H., Qian J., Han C., Sui X., Liu T. (2021). Preparation of Folic Acid-Conjugated Albumin Nanoparticles Containing Paclitaxel Using High-Pressure Homogenisation Coagulation Method. Res. Sq..

[126] Sun Y., Zhao Y., Teng S., Hao F., Zhang H., Meng F., Zhao X., Zheng X., Bi Y., Yao Y. (2019). Folic Acid Receptor-Targeted Human Serum Albumin Nanoparticle Formulation of Cabazitaxel for Tumor Therapy. Int. J. Nanomed..

[127] Alexander C.M., Hamner K.L., Maye M.M., Dabrowiak J.C. (2014). Multifunctional DNA-Gold Nanoparticles for Targeted Doxorubicin Delivery. Bioconjugate Chem..

[128] Gabizon A., Horowitz A.T., Goren D., Tzemach D., Mandelbaum-Shavit F., Qazen M.M., Zalipsky S. (1999). Targeting Folate Receptor with Folate Linked to Extremities of Poly(Ethylene Glycol)-Grafted Liposomes: In Vitro Studies. Bioconjugate Chem..

[129] Aronov O., Horowitz A.T., Gabizon A., Gibson D. (2003). Folate-Targeted PEG as a Potential Carrier for Carboplatin Analogs. Synthesis and In Vitro Studies. Bioconjugate Chem..

[130] Nanda J.S., Lorsch J.R. (2014). Labeling a Protein with Fluorophores Using NHS Ester Derivitization.

[131] Thermo Scientific (2022). Bioconjugation and Crosslinking Technical Handbook.

[132] Bychkova A.V., Gorobets M.G., Toroptseva A.V., Markova A.A., Nguyen M.T., Volodina Y.L., Gradova M.A., Abdullina M.I., Mayorova O.A., Kasparov V.V. (2025). Folate-Modified Albumin-Functionalized Iron Oxide Nanoparticles for Theranostics: Engineering and In Vitro PDT Treatment of Breast Cancer Cell Lines. Pharmaceutics.

[133] Pearson A.J., Roush W.R., Wiley J. (1999). Activating Agents and Protecting Groups.

[134] Starosta R., Santos F.C., Almeida R.F.M. (2020). De Human and Bovine Serum Albumin Time-Resolved Fl Uorescence: Tryptophan and Tyrosine Contributions, Effect of DMSO and Rotational Diffusion. J. Mol. Struct..

[135] Pabbathi A., Patra S., Samanta A. (2013). Structural Transformation of Bovine Serum Albumin Induced by Dimethyl Sulfoxide and Probed by Fluorescence Correlation Spectroscopy and Additional Methods. Chemphyschem.

[136] Batista A.N.L., Batista J.M., Bolzani V.S., Furlan M., Blanch E.W. (2013). Selective DMSO-Induced Conformational Changes in Proteins from Raman Optical Activity. Phys. Chem. Chem. Phys..

[137] Liu R., Qin P., Wang L., Zhao X., Liu Y., Hao X. (2010). Toxic Effects of Ethanol on Bovine Serum Albumin. J. Biochem. Mol. Toxicol..

[138] Lin S., Wei Y., Li M., Wang S. (2004). Effect of Ethanol or/and Captopril on the Secondary Structure of Human Serum Albumin before and after Protein Binding. Eur. J. Pharm. Biopharm..

[139] Kim D., Kim E., Kim J., Park K.M., Baek K., Jung M., Ko Y.H., Sung W., Kim H.S., Suh J.H. (2007). Direct Synthesis of Polymer Nanocapsules with a Noncovalently Tailorable Surface. Angew. Chem.-Int. Ed..

[140] Off M.K., Steindal A.E., Porojnicu A.C., Juzeniene A., Vorobey A., Johnsson A., Moan J. (2005). Ultraviolet Photodegradation of Folic Acid. J. Photochem. Photobiol. B Biol..

[141] Liang L., Zhang J., Zhou P., Subirade M. (2013). Protective Effect of Ligand-Binding Proteins against Folic Acid Loss Due to Photodecomposition. Food Chem..

[142] Yang W., Li Z., Li Y., He W., Yan J. (2024). Transforming Albumin into a Trojan Horse of Immunotherapy-Resistant Colorectal Cancer with a High Microsatellite Instability. ACS Nano.

[143] Haponiuk T., Thomas S., Gopi S., Jacob J. (2018). Biopolymer Based Nanomaterials in Drug Delivery Systems: A Review. Mater. Today Chem..

[144] Mohos V., Fliszár-Nyúl E., Schilli G., Hetényi C., Lemli B., Kunsági-Máté S., Bognár B., Poór M. (2018). Interaction of Chrysin and Its Main Conjugated Metabolites Chrysin-7-Sulfate and Chrysin-7-Glucuronide with Serum Albumin. Int. J. Mol. Sci..

[145] Liu Y., Long M., Xie M.X. (2013). Mechanism of Interaction between Chrysin and Different Configurations of Human Serum Albumin. Wuli Huaxue Xuebao/Acta Phys.-Chim. Sin..

[146] Jiang B., Zhao A., Miao J., Chang P., Chen H., Pan W., Lin C. (2014). Molecular Docking and Reaction Kinetic Studies of Chrysin Binding to Serum Albumin. Nat. Prod. Commun..

[147] Paal K., Shkarupin A., Beckford L. (2007). Paclitaxel Binding to Human Serum Albumin-Automated Docking Studies. Bioorganic Med. Chem..

[148] Trynda-Lemiesz L. (2004). Paclitaxel-HSA Interaction. Binding Sites on HSA Molecule. Bioorganic Med. Chem..

[149] Liu H., Bao W., Ding H., Jang J., Zou G. (2010). Binding Modes of Flavones to Human Serum Albumin: Insights from Experimental and Computational Studies. J. Phys. Chem. B.

[150] Zheng K., Li R., Zhou X., Hu P., Zhang Y., Huang Y., Chen Z., Huang M. (2015). Dual Actions of Albumin Packaging and Tumor Targeting Enhance the Antitumor Efficacy and Reduce the Cardiotoxicity of Doxorubicin in Vivo. Int. J. Nanomed..

[151] Cheng L.Y., Fang M., Bai A.M., Ouyang Y., Hu Y.J. (2017). Insights into the Interaction of Methotrexate and Human Serum Albumin: A Spectroscopic and Molecular Modeling Approach. Luminescence.

[152] Preisz Z., Kunsági-Máté S. (2021). Effect of Methotrexate and Its Photodegradation Products on the Temperature Induced Denaturation of Human Serum Albumin. Spectrochim. Acta-Part A Mol. Biomol. Spectrosc..

[153] Alam P., Abdelhameed A.S., Rajpoot R.K., Khan R.H. (2016). Interplay of Multiple Interaction Forces: Binding of Tyrosine Kinase Inhibitor Nintedanib with Human Serum Albumin. J. Photochem. Photobiol. B Biol..

[154] Ali M.S., Al-Lohedan H.A. (2022). Experimental and Computational Investigation on the Interaction of Anticancer Drug Gemcitabine with Human Plasma Protein: Effect of Copresence of Ibuprofen on the Binding. Molecules.

[155] Rasse-Suriani F.A.O., Costa R.A., Denofrio M.P., Garcia Einschlag F.S., Cabrerizo F.M. (2025). Interaction of Normelinonine F and Related N-Methyl-β-Carbolines Derivatives with Bovine Serum Albumin. Spectroscopic Profiles, Multivariate Analysis and Theoretical Calculations. Int. J. Biol. Macromol..

[156] Molaei P., Mahaki H., Manoochehri H., Tanzadehpanah H. (2022). Binding Sites of Anticancer Drugs on Human Serum Albumin (HSA): A Review. Protein Pept. Lett..

[157] Shen H., Gu Z., Jian K., Qi J. (2013). In Vitro Study on the Binding of Gemcitabine to Bovine Serum Albumin. J. Pharm. Biomed. Anal..

[158] Sarmah S., Pahari S., Das S., Belwal V.K., Jana M., Singha Roy A. (2020). Non-Enzymatic Glycation of Human Serum Albumin Modulates Its Binding Efficacy Towards Bioactive Flavonoid Chrysin: A Detailed Study Using Multi-Spectroscopic and Computational Methods.

[159] Gopi P., Singh S., Islam M.M., Yadav A., Gupta N., Pandya P. (2022). Thermodynamic and Structural Profiles of Multi-Target Binding of Vinblastine in Solution. J. Mol. Recognit..

[160] Chatterjee S., Dube A., Majumder S.K. (2025). Unveiling Role of Serum Albumin in Disaggregation and Cellular Delivery of a near—Infrared Chlorophyll—Based Photosensitizer in Breast Cancer Cells. Photochem. Photobiol. Sci..

[161] Marconi A., Mattioli E.J., Ingargiola F., Giugliano G., Marforio T.D., Prodi L., Giosia M.D., Calvaresi M. (2023). Dissecting the Interactions Between Chlorin E6 and Human the Interactions Between Chlorin E6 and Human. Molecules.

[162] Beltukova D.M., Belik V.P., Chudakov K.A., Smirnov O.V., Semenova I.V. (2025). Time-Resolved Phosphorescence Analysis of Singlet Oxygen Generation and Radachlorin/Ce6 Triplet State Quenching in Solutions with Albumin. Chem. Phys. Lett..

[163] Zhang Y., Go H. (2009). Dyes and Pigments Photoprocesses of Chlorin E6 Bound to Lysozyme or Bovin Serum Albumin. Dye. Pigment..

[164] Li X., Li X., Park S., Wu S., Guo Y., Taek K., Kwon N., Yoon J., Hu Q. (2024). Photodynamic and Photothermal Therapy via Human Serum Albumin Delivery. Coord. Chem. Rev..

[165] Zarubaev V.V., Kris’kO T.C., Kriukova E.V., Muraviova T.D. (2017). Effect of Albumin on the Fluorescence Quantum Yield of Porphyrin -Based Agents for Fluorescent Diagnostics. Photodiagnosis Photodyn. Ther..

[166] Siddiqui S., Ameen F., ur Rehman S., Sarwar T., Tabish M. (2021). Studying the Interaction of Drug/Ligand with Serum Albumin. J. Mol. Liq..

[167] Lee C., Kang S. (2021). Development of HER2-Targeting-Ligand-Modified Albumin Nanoparticles Based on the SpyTag/SpyCatcher System for Photothermal Therapy. Biomacromolecules.

[168] Ekinci M., Alencar L.M.R., Lopes A.M., Santos-Oliveira R., İlem-Özdemir D. (2023). Radiolabeled Human Serum Albumin Nanoparticles Co-Loaded with Methotrexate and Decorated with Trastuzumab for Breast Cancer Diagnosis. J. Funct. Biomater..

[169] Baneshi M., Dadfarnia S., Shabani A.M.H., Sabbagh S.K., Haghgoo S., Bardania H. (2019). A Novel Theranostic System of AS1411 Aptamer-Functionalized Albumin Nanoparticles Loaded on Iron Oxide and Gold Nanoparticles for Doxorubicin Delivery. Int. J. Pharm..

[170] Karmali P.P., Kotamraju V.R., Kastantin M., Black M., Missirlis D., Tirrell M., Ruoslahti E. (2009). Targeting of Albumin-Embedded Paclitaxel Nanoparticles to Tumors. Nanomed. Nanotechnol. Biol. Med..

[171] Look J., Wilhelm N., Von Briesen H., Noske N., Günther C., Langer K., Gorjup E. (2015). Ligand-Modified Human Serum Albumin Nanoparticles for Enhanced Gene Delivery. Mol. Pharm..

[172] Jiang M., Li S., Wu J., Li W., Wen X.A., Liang H., Yang F. (2021). Designing Biotin-Human Serum Albumin Nanoparticles to Enhance the Targeting Ability of Binuclear Ruthenium(III) Compound. J. Inorg. Biochem..

[173] Solanki R., Patel S. (2023). Preparation, Characterization and In Vitro Anticancer Efficacy of Biotin-Conjugated, Silibinin Loaded Bovine Serum Albumin Nanoparticles. Food Biosci..

[174] Kang M.S., Kong T.W.S., Khoo J.Y.X., Loh T.P. (2021). Recent Developments in Chemical Conjugation Strategies Targeting Native Amino Acids in Proteins and Their Applications in Antibody-Drug Conjugates. Chem. Sci..

[175] Stefanie M., Bich C., Touboul D., Zenobi R. (2009). Chemical Cross-Linking with NHS Esters: A Systematic Study on Amino Acid Reactivities. J. Mass Spectrom..

[176] Varanko A., Saha S., Chilkoti A. (2020). Recent Trends in Protein and Peptide-Based Biomaterials for Advanced Drug Delivery. Adv. Drug Deliv. Rev..

[177] Patil U.S., Qu H., Caruntu D., O’Connor C.J., Sharma A., Cai Y., Tarr M.A. (2013). Labeling Primary Amine Groups in Peptides and Proteins with N-Hydroxysuccinimidyl Ester Modified Fe_3_O_4_@SiO_2_ Nanoparticles Containing Cleavable Disulfide-Bond Linkers. Bioconjugate Chem..

[178] Stukan I., Żuk A., Pukacka K., Mierzejewska J., Pawłowski J., Kowalski B., Dąbkowska M. (2025). Wolf in Sheep’s Clothing: Taming Cancer’s Resistance with Human Serum Albumin?. Int. J. Nanomed..

[179] Panja S., Khatua D.K., Halder M. (2018). Simultaneous Binding of Folic Acid and Methotrexate to Human Serum Albumin: Insights into the Structural Changes of Protein and the Location and Competitive Displacement of Drugs. ACS Omega.

[180] Al Jaseem M.A.J., Abdullah K.M., Qais F.A., Shamsi A., Naseem I. (2021). Mechanistic Insight into Glycation Inhibition of Human Serum Albumin by Vitamin B9: Multispectroscopic and Molecular Docking Approach. Int. J. Biol. Macromol..

[181] Usacheva T., Gamov G., Bychkova A., Anufrikov Y., Shasherina A., Alister D., Kuranova N., Sharnin V. (2022). Binding of Quercetin and Curcumin to Human Serum Albumin in Aqueous Dimethyl Sulfoxide and in Aqueous Ethanol. J. Therm. Anal. Calorim..

[182] Markarian S.A., Aznauryan M.G. (2012). Study on the Interaction between Isoniazid and Bovine Serum Albumin by Fluorescence Spectroscopy: The Effect of Dimethylsulfoxide. Mol. Biol. Rep..

[183] Zhang H., Bao Y., Li G., Li S., Zhang X., Guo C., Wu X., Jin Y. (2024). PH-Responsive Hyaluronic Acid Nanomicelles for Photodynamic/Chemodynamic Synergistic Therapy Trigger Immunogenicity and Oxygenation. ACS Biomater. Sci. Eng..

[184] Krueger A.T., Imperiali B. (2013). Fluorescent Amino Acids: Modular Building Blocks for the Assembly of New Tools for Chemical Biology. ChemBioChem.

[185] Kashkanova A.D., Albrecht D., Küppers M., Blessing M., Sandoghdar V. (2024). Measuring Concentration of Nanoparticles in Polydisperse Mixtures Using Interferometric Nanoparticle Tracking Analysis. ACS Nano.

[186] Bauer I.A., Dmitrienko E.V. (2024). Investigating Non-Covalent Interactions of Human Serum Albumin with Doxorubicin and Folic Acid. Biochem. Suppl. Ser. B Biomed. Chem..

